# Direct and indirect effects of agricultural land cover on avian biodiversity in eastern Canada

**DOI:** 10.1007/s10531-023-02559-1

**Published:** 2023-03-04

**Authors:** Michelle Rabbetts, Lenore Fahrig, Greg W. Mitchell, Kevin C. Hannah, Sara J. Collins, Scott Wilson

**Affiliations:** 1 Department of Biology, Carleton University, Ottawa, Canada; 2 Wildlife Research Division, Environment and Climate Change Canada, Ottawa, ON Canada; 3 Canadian Wildlife Service, Environment and Climate Change Canada, Ottawa, ON Canada; 4 Department of Forest and Conservation Sciences, University of British Columbia, Vancouver, BC Canada; 5 Wildlife Research Division, Environment and Climate Change Canada, Delta, BC Canada

**Keywords:** Agricultural expansion, Bird, Forest loss, Species richness, Structural equation modeling

## Abstract

Agriculture is one of the largest threats to global biodiversity. However, most studies have focused only on the direct effects of agriculture on biodiversity, and few have addressed the indirect effects, potentially over or under-estimating the overall impacts of agriculture on biodiversity. The indirect effect is the response not to the agricultural cover types or operations *per se*, but instead, to the way that agriculture influences the extent and configuration of different types of natural land cover in the landscape. We used structural equation modelling (SEM) to evaluate the direct, indirect, and total effects of agriculture on species richness of three bird guilds: forest birds, shrub-edge birds, and open country birds. We found that forest bird richness was driven by the negative indirect effect of cropland via forest loss. Shrub-edge and open country bird richness increased with the amount of agriculture land covers; however, importantly, we found negative indirect effects of agriculture on both guilds via a reduction in more natural land covers. This latter result highlights how we would have over-estimated the positive effects of agriculture on shrub-edge and open country bird richness had we not measured both direct and indirect effects (i.e., the total effect size is less than the direct effect size). Overall, our results suggest that a bird-friendly agricultural landscape in our region would have forest that is configured to maximize forest edge, and a high proportion of perennial forage within the agricultural portion of the landscape.

**Supplementary Information:**

The online version contains supplementary material available at 10.1007/s10531-023-02559-1.

## Introduction

Agriculture is one of the largest threats to global biodiversity and has been shown to impact the diversity and abundance of a range of taxa in temperate and tropical zones (Dirzo and Raven 2003; Devictor and Jiguet 2007; Tilman et al. 2017; Stanton et al. 2018; Martin et al. 2020). While there has been considerable research on the impacts of agriculture, we know less about whether these impacts are direct or indirect. Direct effects imply a direct relationship between agriculture and species response and could either be a response to the agricultural land cover or to agricultural operations within agricultural land (e.g., chemical application, tilling, mowing crops). Indirect effects of agriculture in this context refer to a response to how the activity of agriculture transforms the landscape, beyond the insertion of agricultural land cover (e.g., loss of natural land covers like forest or shrubland). In this case, the response is not to the agricultural cover types or operations *per se*, but instead, to the way that agriculture influences the extent and configuration of different types of natural land cover in the landscape.

Whether agriculture influences species directly or indirectly has implications for how we might manage agricultural landscapes. If agriculture itself is directly influencing species, then our efforts should target the agricultural component of the landscape, by manipulating the agricultural cover types or the activities on agricultural land (e.g., chemical application, tilling). On the other hand, if agriculture is indirectly influencing species through its effect on the availability of more natural or less intensively managed land covers, then our efforts should focus on how to best manage the composition and configuration of more natural landscape components within the agroecosystem. In many cases both direct and indirect effects will likely influence species, but by understanding their relative impacts we can determine how management strategies might be best directed as well as a more accurate understanding of how different agricultural land covers affect diversity via the assessment of total effect sizes.

Structural equation modeling (SEM) is an analytical approach that allows for the joint evaluation of the direct and indirect effects of landscape change on biodiversity (Lefcheck 2019). SEM combines multiple relationships among variables in a single model and has been used widely in ecological research (reviewed in Fan et al. 2016). For example, SEM has been used to determine the indirect effects of habitat loss on biodiversity through its effect on habitat fragmentation (Püttker et al. 2020), and the indirect effects of agricultural landscape pattern on aquatic invertebrate diversity through its effects on water chemistry (Collins et al. 2019).

In this study, we used SEM to evaluate the direct and indirect effects of agriculture on bird species richness in an historically forested region in eastern Canada. Several studies have examined how the expansion of agriculture impacts species diversity in this ecosystem. Where native grasslands are rare or absent, the presence of agriculture creates open habitats that is beneficial for open country breeding birds, although there is intraspecific variation in the use of agricultural lands within the open country bird guild (Frei et al. 2018; de Zwaan et al. 2022). The presence of field edges and hedgerows (i.e., shrubby field margins) within agricultural lands can also provide breeding habitat and connectivity among patches for shrub and forest edge breeding species that prefer semi-open landscapes (Benton et al. 2003; Fonderflick et al. 2013; Wilson et al. 2017). In other words, shrub and forest edge birds can benefit from the openness of agriculture as long as suitable shrub and edge habitats are present in the landscape (Wilson et al. 2017). For obligate forest breeding birds, the openness created by agricultural expansion into forested areas typically results in a loss of habitat and a decline in species diversity driven most strongly by the loss of foliage-gleaners and insectivores (Endenburg et al. 2019).

Our overarching goals were to identify the strength of direct and indirect effects of agricultural expansion and how those respective effects differ across guilds of forest, shrub-forest edge (hereafter ‘shrub-edge’), and open country breeding birds. Overall, we expect the direct effect of agriculture to influence bird species richness, but because agricultural land comes at the expense of natural land covers, we also expect that agriculture will indirectly affect species richness by reducing the availability of their natural habitats. To assess these effects, we measured agricultural variables (cropland, perennial forages and grassland, and mean field size) and natural land cover variables (forest, shrubland, hedgerows, and forest edge) in the landscape surrounding each site. We made *a priori* predictions about relationships between these landscape variables and the richness of each guild (Table 1). We then used these predictions to develop three SEM models, one for each bird guild, to estimate the direct and indirect effects of agriculture on guild richness. To examine if relationships for species of conservation concern are similar to those observed for the full bird community, we also conducted a separate analysis using only the species within each guild that have shown long-term population declines.

**Table 1:** Hypotheses for *a priori* predicted relationships in forest, open country and shrub-edge bird structural equation modelling diagrams. Positive predicted relationships are represented by +, negative predicted relationships are represented by –, and peaked predicted relationships are represented by ∩

Relationship	Direction	Hypothesis	References
Cropland → Mean Field Size	+	As cropland increases, mean field size increases because agricultural (i.e. cropland) expansion and intensification frequently include the amalgamation of smaller fields to larger fields.	Benton et al., 2003; Wilson et al., 2017
Cropland → Perennial Forages and Grassland	*–*	As cropland increases, perennial forages and grassland decrease because perennial forages and grassland may be converted to cropland to increase agriculture productivity.	Barretto et al., 2013; Stanton et al., 2018
Cropland → Forest AND Cropland → Shrubland	*–*	As cropland increases, other natural covers such as forest and shrubland decrease because the expansion of cropland comes at the expense of other land covers.	Wilson et al., 2017; Endenburg et al., 2019; Santana et al., 2017; Frei et al., 2018; Stanton et al., 2018
Perennial Forages and Grassland → Forest AND Perennial Forages and Grassland → Shrubland	*–*	As perennial forages and grassland increases, other natural covers such as forest and shrubland decrease because the expansion of perennial forages comes at the expense of other natural land covers.	Wilson et al., 2017; Endenburg et al., 2019; Santana et al., 2017; Stanton et al., 2018
Mean Field Size → Hedgerow	*–*	As mean field size increases, hedgerow amount decreases because field edges are lost between fields.	Benton et al., 2003; Šálek et al., 2018
Mean Field Size → Open Country Bird Richness	+	As mean field size increases, open country bird richness increases because nest predation is highest at field edges. Large fields have a lower edge-to-area ratio, reducing overall nest predation on open country birds.	Herkert et al., 2003
Perennial Forages and Grassland → Open Country Bird Richness	+	As perennial forages and grassland increase, open country bird richness increases because perennial forages provides nesting habitat mimicking native grassland.	Herkert et al., 2003; Frei et al., 2018
Forest → Forest Bird Richness	+	As forest increases, forest bird richness increases because forest provides nesting habitat.	Rodewald and Yahner, 2001; Endenburg et al. 2019
Shrubland → Shrub-Edge Bird Richness	+	As shrubland increases, shrub-edge bird richness increases because shrubland provides nesting habitat.	Nikolov et al., 2011; Shake et al., 2011
Hedgerow → Forest Bird Richness	+	As hedgerows increase, forest bird richness increases because hedgerows provide nesting and roosting habitat, and connectivity among forest patches.	Benton et al., 2003; Wilson et al., 2017
Hedgerow → Open Country Bird Richness	*–*	As hedgerows increase, open country bird richness decreases because birds nesting near hedgerows suffer high predation rates.	Herkert et al., 2003; Wilson et al., 2017; Stanton et al., 2018
Hedgerow → Shrub-Edge Bird Richness	+	As hedgerows increase, shrub-edge bird richness increases because hedgerows provide nesting and roosting habitat.	Benton et al., 2003; Wilson et al., 2017
Forest → Forest Edge	∩	Forest edge amount is highest at moderate amounts of forest because low amounts of forest have less perimeter or edge and high amounts of forest amalgamate into fewer patches that result in less forest perimeter or edge.	
Forest Edge → Shrub-Edge Bird Richness	+	As forest edge increases, shrub-edge bird richness increases because forest edge provides nesting habitat.	Fonderflick et al., 2013

## Methods

### Overview

**Fig. 1:**
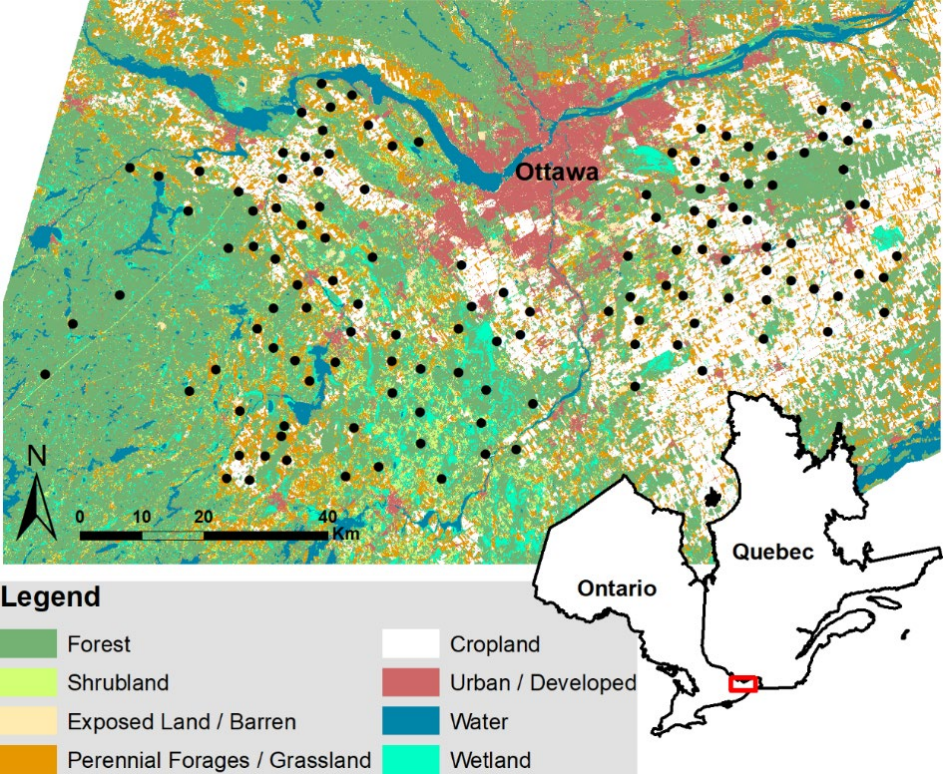
Map showing the locations of 127 bird survey sites near Ottawa, Ontario, Canada with land cover types from the 2016 Agriculture and Agri-Food Canada annual crop inventory

We sampled birds using autonomous recording units at 127 sites along roads in eastern Ontario during the 2016 breeding season (Fig. 1). We sampled at three time periods in the early morning over 2 days per site to determine bird richness (number of species) for forest, shrub-edge, and open country breeding birds. In the landscape around each site, we measured mean field size, the length of forest edge and the proportion of the landscape in cropland, perennial forages and grassland, forest, shrubland, and hedgerows. We created three global structural equation models representing our predictions for the three bird guilds (Supplemental Material Figure S7-S9). Using confirmatory path analysis, we tested the relationships among the agricultural land cover variables, natural and less intensely managed land cover variables, and bird richness. We then summarized the direct effects of agricultural land cover, indirect effects of agriculture on natural and less intensively managed land covers, and the total agricultural effect (i.e., the summation of the direct and indirect effects) on bird richness.

### Site selection

We used a GIS road layer (Esri Inc. 2016) to randomly place potential bird sampling sites on accessible roads, except for primary highways. This resulted in 205 potential sites, of which we identified 127 that represented gradients along two land cover axes within 1 km of the sites: (1) a high proportion of agriculture to a high proportion of forest and (2) within the agricultural land cover class, a high proportion of cropland to a high proportion of pastureland and forage crops. Land cover types were identified using the Agriculture and Agri-Food Canada annual crop inventory (Fisette et al. 2013).

### Bird sampling methods

We conducted surveys of the bird communities from 6 June to 16 July 2016 at the 127 sites (Fig. 1). This window was selected to exclude migratory species that may still be moving through the sites in late May while also including species with longer breeding periods that extend into mid-July. We used SM2 + autonomous recording units (ARUs; Wildlife Acoustics Inc.) placed at approximate breast height on a tree along the roadside, with the microphones oriented perpendicular to the road, to make acoustic recordings. We used a sampling rate of 24 kHz or double the maximum frequency of the typical songs of most birds in this region, and a bit depth of 16 bits. We tested microphone sensitivity prior to ARU deployments to ensure comparable signal-to-noise ratio and detection radius between units (Turgeon et al. 2017; Darras et al. 2020). The sampling radius for species observations was 250 m and remained consistent across all sites. The ARUs were originally programmed to record for 10 min every half hour from 60 min before sunrise until 4 h after sunrise for a seven-day period. We selected a systematic subsample of these recordings for interpretation, using the first and last clear (i.e., no heavy rain or wind) recording dates (generally 5–7 days apart) at each site. Within selected dates, a single skilled observer interpreted the first 3 min of each 10-minute recording for three time periods: 30 min before sunrise, 30 min after sunrise, and 90 min after sunrise.

### Bird guilds

We assigned each species to one of four guilds based on its breeding habitat associations provided in the Birds of North America Online (Rodewald 2015): forest, shrub-edge, open country, and wetland guilds. Forest species are associated with forest habitat. Shrub-edge species are associated with shrub, scrub, early successional, and forest edge habitats. Open country species are associated with grassland and/or open agricultural field habitats. Although species were initially assigned to wetland guilds, there were very few obligate wetland species after the classification was completed, likely owing to the lack of natural wetland habitat in our study area. Therefore, we did not include wetland species in this analysis. Guild richness at each site was calculated as the number of species present for each guild (Supplementary Material, Table S2).

We used long-term (1970 to 2019) population trends in Canada from the North American Breeding Bird Survey (BBS, Smith et al. 2020) to identify whether direct and indirect effects of agriculture are similar for species of conservation concern as they are for the broader bird community. The BBS is a volunteer-based roadside survey that was initiated in 1966 and is the primary source of information on continental trends of North American birds. We classified species of conservation concern as those showing significant negative long-term population trends based on mean and 95% credible intervals below 0. After this group of species was identified, we conducted the same confirmatory path analysis as for all species (Supplemental Material Table S4).

### Landscape variables

We calculated the amount of cropland, perennial forages and grassland, forest, shrubland, hedgerows, mean field size, and forest edge length within a 1 km^2^ square around each bird survey site using the 2016 Agriculture and Agri-Food Canada annual crop inventory (AAFC 2016; Fisette et al. 2013). This dataset has a 30-m resolution and includes 66 land cover classes (Fisette et al. 2013). The 1 km^2^ scale is similar to the size of many farms in the study region and is therefore a relevant scale for farmland management applications. The 1 km^2^ scale was used only to measure the landscape variables and the scale remained consistent across all sites and for all variables.

We grouped land cover types to create forest, cropland, and perennial forages/grassland land covers. The forest land cover combined mixed-wood, broadleaf, and coniferous forest. For cropland we combined 20 classes of annual row crops, primarily corn, soy, wheat, and vegetables and fruits. Perennial forages included pasture and hay (Statistics Canada 2011). We combined grassland with perennial forages, because native grassland was uncommon in our study region and perennial forages functionally mimic grassland habitat for birds (Santana et al. 2017; Frei et al. 2018). Hereafter, we refer to this variable as perennial forages and grassland land cover. In addition to the landscape variables, we considered measuring land cover within 150 m of the sample sites to represent the local land cover at the sample site. However, local and landscape (1 km^2^) land covers were highly correlated (Supplemental Material S14 and S15). Therefore, we only used the landscape (1 km^2^) variables in our statistical analyses.

We calculated mean field size, forest edge, and proportion of hedgerows in each landscape using ESRI ArcMap (Esri Inc. 2020a) and aerial imagery (Esri Inc. 2020b). We used Patch Analyst 5 extension (Rempel et al. 2012) to calculate mean field size and total forest edge. Mean field size was calculated for all agricultural land covers. We excluded field fragments by removing all fields that were smaller than 1 ha. Forest edge was calculated as total length of forest edge. To calculate the proportion of a landscape in hedgerows we digitized woody hedgerows using 2020 World Imagery Basemap in ESRI ArcMap (Esri Inc. 2020b), which provides one meter or better resolution satellite and aerial imagery. For digitizing purposes, hedgerows were defined as woody linear strips with agricultural fields on either side that were less than 30 m wide, had no gaps in the canopy greater than 12 m (i.e., average tree canopy size for our region), and were at least 24 m long (i.e., two average tree canopy widths). Last, given that the hedgerow layer was created using imagery from 2020, we also visually compared hedgerows in the 2020 basemap to 2016 Google imagery (Google Earth Pro 2016) to ensure that a hedgerow was not missed because it was removed between 2016 and 2020.

### Statistical analysis

We calculated the direct and indirect relationships between the agricultural variables, natural land cover variables, and bird richness using confirmatory path analysis. We created three structural equation modeling (SEM) diagrams (Shipley 2002, 2009), one for forest, shrub-edge, and open country birds (Supplemental Material Figure S7 – S9). A SEM diagram places variables in a single causal network to test predictions simultaneously. A variable can be both a predictor and response. Note, our modelling approach does not allow a variable to indirectly influence itself via a feedback loop or allow for the inclusion of latent variables. We used confirmatory path analysis because it can easily accommodate small to moderate sample sizes and count data (i.e., richness; Lefcheck 2019). We standardized all variables, except for bird richness, before conducting the analysis. We tested for spatial autocorrelation in species richness for each guild using Moran’s I and the ape package in R (R Core Team 2020).

We conducted confirmatory path analysis in R using the piecewiseSEM package (R Core Team 2020). We began by conducting the directional separation test (Shipley 2002; Gonzalez-Voyer and von Hardenberg 2014), which evaluates the assumption that the hypothesized SEM diagram structure reflects the data, by testing the implied independence between every pair of variables that are assumed not to be directly linked. We used linear and generalized linear models to determine the probability that each unlinked pair was statistically independent (Supplemental Material, Table S9 – S11). We then added links between variables that were found not to be statistically independent, and conducted the directional separation test for the revised models using Fisher’s C statistic:

1 $$C= -2{\sum }_{i=1}^{k}(ln\left({p}_{i}\right))$$

where k is the number of independence claims and *p*_*i*_ is the null probability of the independence test associated with the *i*th independence claim (Shipley 2009). We compared the C value to a chi-square distribution with 2k degrees of freedom (Shipley 2002).

**Fig. 2:**
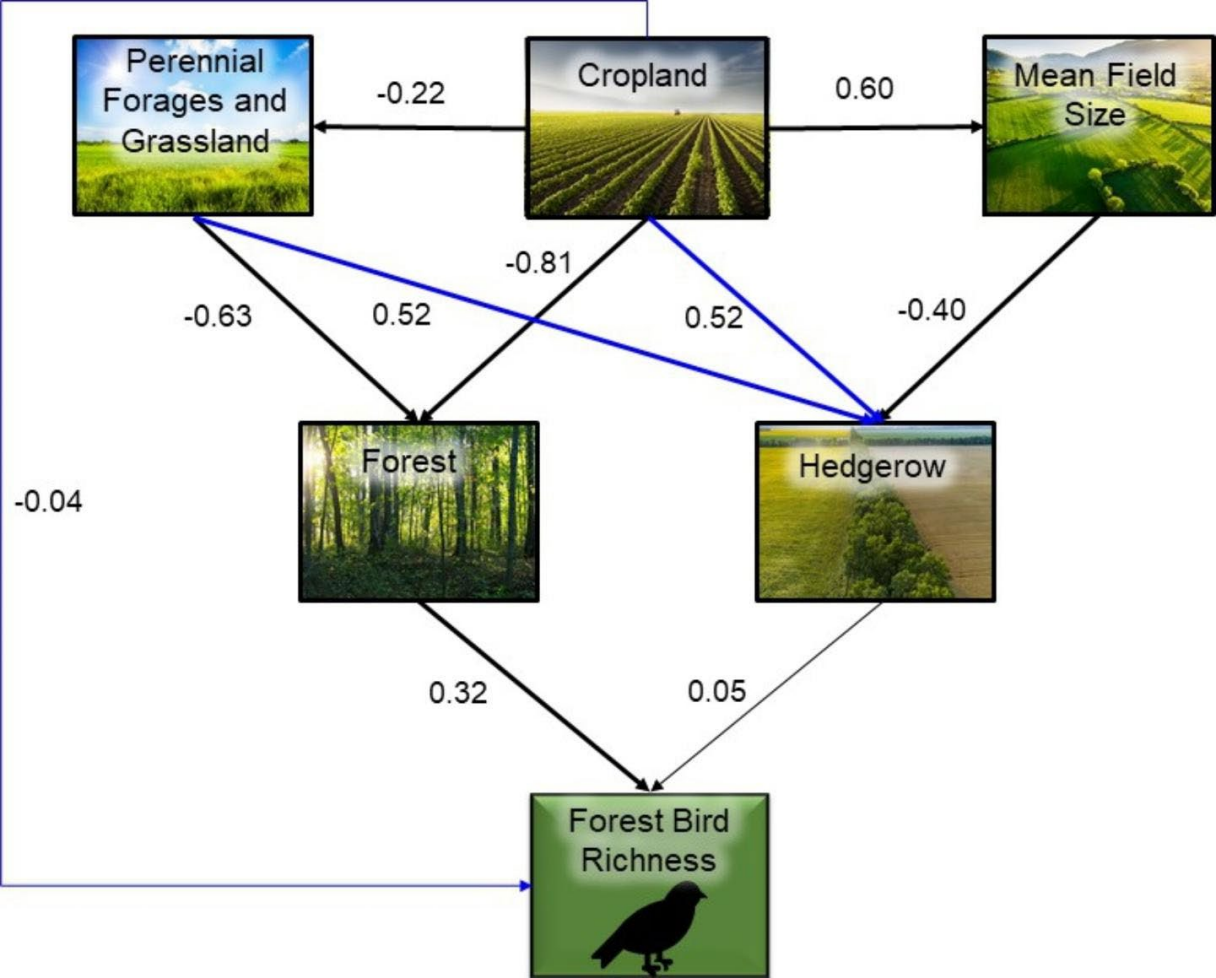
Forest bird structural equation modelling (SEM) diagram with estimates for relationships between agricultural variables, more natural land cover variables, and forest bird richness. Paths with strong statistical support are represented by thick solid links and paths with weak support are represented by thin solid links. Blue links represent relationships not predicted *a priori*

**Fig. 3:**
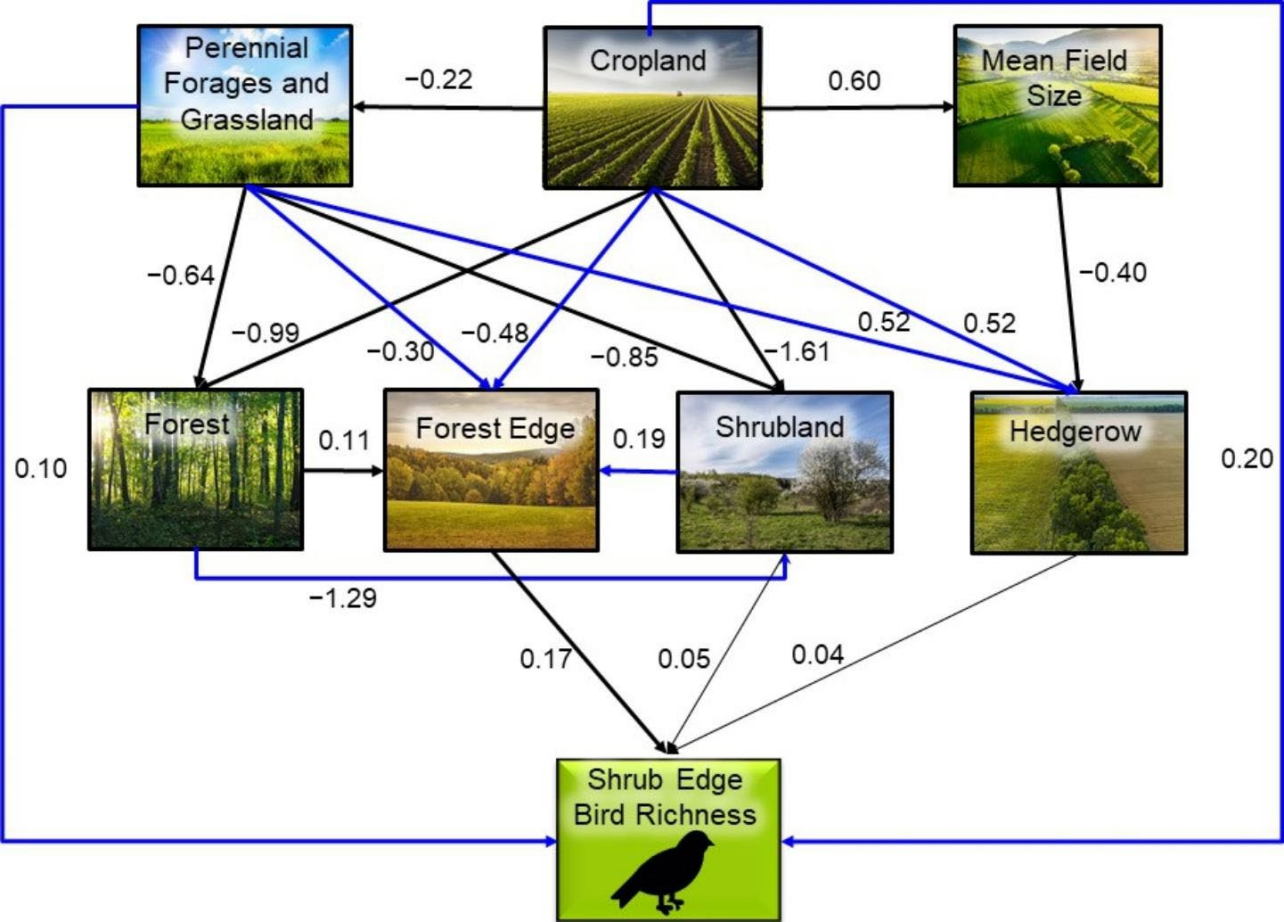
Shrub-edge bird structural equation modelling (SEM) diagram with estimates for relationships between agricultural variables, more natural land cover variables, and shrub-edge bird richness. Paths with strong statistical support are represented by thick solid links and paths with weak support are represented by thin solid links. Blue links represent relationships not predicted *a priori*

**Fig. 4:**
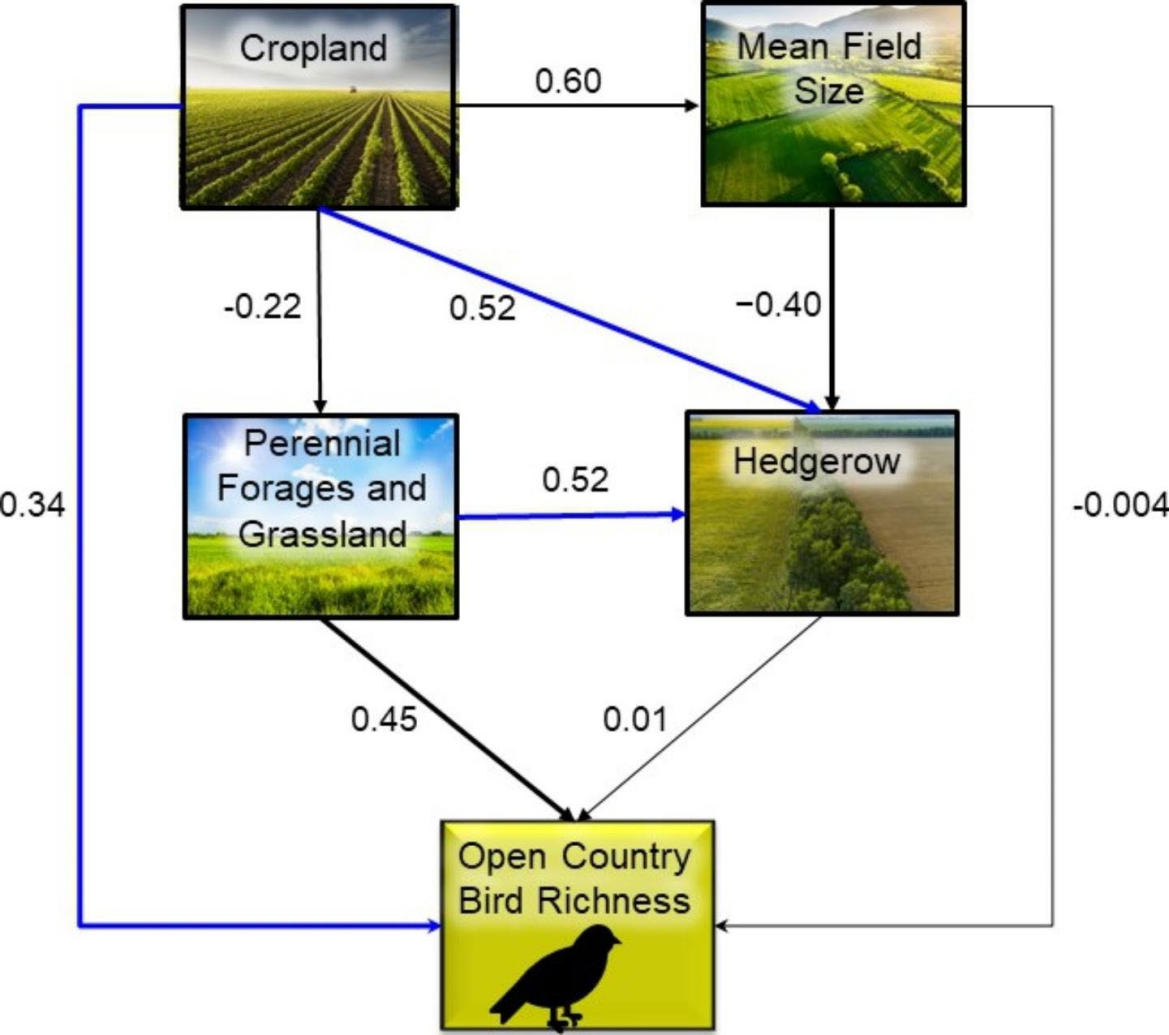
Open country bird structural equation modelling (SEM) diagram with estimates for relationships between agricultural variables, more natural land cover variables, and open country bird richness. Paths with strong statistical support are represented by thick solid links and paths with weak support are represented by thin solid links. Blue links represent relationships not predicted *a priori*

We then fitted a series of linear and generalized linear models for each response variable, including those that were both predictors and responses, to determine the coefficients of all hypothesized paths leading to each response variable in the revised SEM diagrams (Figs. 2, 3 and 4). Bird richness was modeled as count data using a generalized linear model with a Poisson distribution and log-link function. All other variables were modeled using linear models except the effect of forest on forest edge, which was modelled as a quadratic term because we expect a peaked relationship, with low edge both in landscapes with very low forest cover and those with very high forest cover (Fahrig 2003). The effect of forest on forest edge was then calculated by summing the linear and quadratic estimates. For each model we used a model selection approach (Burnham and Anderson 2002) to evaluate the statistical support for individual hypothesized paths within the model. The candidate model sets included the global model containing all predictors hypothesized to directly influence the response, an intercept-only (null) model, and all sub-models derived from the global model. The predictors in the top model with the lowest AICc were considered to have strong support and those in models within 2 AICc units of the top model, but not included in the top model, were considered to have weak support. We tested model fit of the top model by comparing the fitted versus residual values.

We used the parameterized SEM diagrams (Figs. 2, 3 and 4) to determine the direct, indirect, and total effects of the agriculture variables on bird richness. A direct effect was the coefficient of the direct link from an agriculture variable to bird richness. An indirect effect was the product of the coefficients of the links leading from an agriculture variable to bird richness through a more natural land cover variable. The total effect of each agricultural variable on bird richness was the sum of the direct and indirect effects.

## Results

### Bird community

We identified 86 bird species: 48 forest species, 26 shrub-edge species, and 12 open country species (Supplemental Material, Figure S13). The average number of species observed per site was 17 (range 1–28), with an average of 7 shrub-edge species (range: 0–14), 7 forest species (range: 0–18) and 1 open country species (range: 0–5) (Supplemental Material, Table S2). All ubiquitous species were shrub-edge species; American Crow (*Corvus brachyrhynchos*), American Goldfinch (*Spinus tristis*), American Robin (*Turdus migratorius*), and Song Sparrow (*Melospiza melodia*) were recorded at more than 100 sites (Supplemental Material, Table S1). There were 31 species identified as species of conservation concern based on significant negative long-term (1970–2019) population trends from the North American Breeding Bird Survey. The trends for this group ranged from − 21 to -89 across species with a mean of -51 (Supplemental Material, Table S4).

### Spatial autocorrelation

We tested spatial autocorrelation in species richness and found that none of the bird guilds showed spatial autocorrelation, suggesting that across the study region no landscape-scale spatial autocorrelation was influencing bird species richness. More specifically, for each guild, no significant relationship was observed for Moran’s I for the species richness variable. Again, this result suggests that adjacent sites did not share similar richness values and are spatially independent.

### Landscape composition

Forest was the most common land cover in the 1 km^2^ landscapes surrounding the sample sites (average proportion: 0.35, range: 0.03–0.90), followed by cropland (avg: 0.26, range: 0–0.88), perennial forages and grassland (avg: 0.19, range: 0–0.79), shrubland (avg: 0.07, range: 0–0.41), and hedgerow (avg: 0.01, range: 0–0.07). Mean field size was 12.8 ha (range: 0–88.2 ha; note, 7 landscapes had no agricultural fields), and the average total length of forest edge was 8344.9 m (range: 352.4–16839.3 m; Supplemental Material, Table S3). Forest edge length was strongly correlated with the proportion cropland (-0.74), and the proportion forest (0.72; Supplemental Material, Table S5).

### Structural equation modelling (SEM)

Our independence tests (Supplemental Material, Table S9 – S11) suggested that we should add linkages for (1) cropland – hedgerow, cropland – forest edge, and cropland – bird richness for all guilds; (2) perennial forages and grassland – hedgerow, and perennial forages and grassland – forest edge for shrub-edge birds; (3) forest – shrubland for shrub-edge birds; and (4) shrubland – forest edge for shrub-edge birds (Figs. 2, 3 and 4). The Fisher’s C statistics indicated that the correlation structure in the data did not differ from the correlational structure for all three revised path models.

Generally, we correctly predicted the direction of effect for the links in our hypothesized SEM diagrams (Figs. 2, 3 and 4; Table 1; Supplemental Material, Figure S7 – S9). As expected, the amount of agriculture (cropland, perennial forages and grassland) reduced all woody and shrubby land cover variables (i.e., forest, shrubland, and forest edge), but had a relatively strong positive effect on hedgerow amount. Also consistent with our predictions, cropland was positively related to mean field size and mean field size was negatively related to hedgerow amount. The richness of bird guilds responded positively to the natural and less intensively managed land covers in the predicted directions except for open country bird richness, which responded slightly positively to hedgerow cover, although the effect was very small. There were also some direct responses to agriculture variables. Among the direct effects between agriculture and richness, we found that as predicted, shrub-edge and open country bird richness increased with both the proportion of cropland and the proportion of perennial forages and grassland (Supplemental Material, Figure S2 – S3, S5 - S6). As predicted, shrub-edge bird richness increased in landscapes with more forest edge and forest bird richness increased with forest amount (Supplemental Material, Figure S1, S5).

Indirect effects of the agriculture variables varied with bird guild. Overall, the indirect effects of agriculture were predominantly negative across guilds. For forest bird richness, the indirect effects of agriculture drove the overall negative effect. In other words, the direct effect of cropland on forest bird richness was weakly negative; however, there was a strong negative relationship between cropland on forest amount, resulting in a strong negative indirect effect (Table 2; Fig. 2). The one exception to the negative indirect effect of agriculture on richness across guilds was a positive indirect effect of cropland on shrub-edge birds via the negative effect of cropland on forest, a negative effect of forest on shrubland amount, a positive effect of shrubland amount on forest edge amount, and the positive effect of forest edge on richness (Table 2; Fig. 3). While the indirect effect of cropland on open country bird richness was negative, the direct effect was positive, resulting in an overall positive effect. Mean field size, unexpectedly, had a weak negative total effect on open country bird richness (Table 2; Fig. 4).

**Table 2:** Summary of the direct, indirect, and total effects of agriculture variables (cropland, perennial forages and grassland, and mean field size) on bird richness (forest, shrub-edge and open country bird richness). Direct effects are derived from the direct relationship (i.e. link) between the agricultural variable and bird richness. Indirect effects were derived from the sum of all indirect pathways leading to bird richness from the agricultural variable, where each pathway is the product of the estimates for all relationships (i.e. link) in the pathway. The proportional change to the total effect is relative to the direct effect, where - represents negative and + represents positive

Bird Richness Guild	Agriculture Variable	Indirect Pathways (number)	Direct Effect	Indirect Effect(s)	Total Effect	Proportional change relative to direct effect
Forest	Cropland	5	-0.04	-0.207	-0.247	6.2 X more -
Shrub-Edge	Cropland	15	0.20	0.065	0.265	1.3 X more +
Shrub-Edge	Perennial Forages and Grassland	7	0.10	-0.044	0.056	0.6 X less +
Open Country	Cropland	5	0.34	-0.100	0.240	0.7 X less +
Open Country	Mean Field Size	1	-0.004	-0.004	-0.008	2.0 X more -

Comparisons of direct and indirect effects for declining species relative to all species showed only slight differences for forest and open country bird guilds but a few more modest changes for the shrub-edge guild (compare Table 2, S8). In particular, relative to all shrub-edge bird species we found an increased positive direct effect of perennial forages and grassland and a change from a positive to a slightly negative indirect effect of cropland for shrub-edge birds that are in decline. We also found a reduced influence of forest-edge and a greater influence of shrubland for the declining subset of shrub-edge species relative to all species (see Figs. 2, 3 and 4 and S10-S12 for comparisons of individual linkages).

## Discussion

In this study, we separated the direct and indirect effects of agriculture to derive total effect sizes of agriculture on avian richness, which allowed us to more accurately interpret how to manage agricultural landscapes. By separating the direct and indirect effects, we can better understand the complex relationship between agriculture and bird richness including effects that operate indirectly when agriculture results in a loss of natural land cover. For example, while unsurprisingly the effect of agriculture on forest birds was strongly negative, our results revealed the effect was primarily driven indirectly by forest loss. In the case of open country birds, row crops have both a direct benefit but an indirect cost because they lead to a loss of grasslands and perennial forages. The net result of this interaction is that the total effect of agriculture, while positive, is less than expected based on the direct effect of row crops or perennial forages and grasslands when each are assessed independently.

Our results suggest that the negative effect of cropland on forest bird richness is not driven by agriculture itself, but rather by the negative relationship between cropland and forest (i.e., the loss of habitat for forest bird species). While agriculture has a strong negative total effect on forest birds, the effect is more than six times more negative than the direct effect. Evidently, agriculturally driven forest loss is the strongest driver of forest birds’ relationship with agriculture. Our study region was predominantly forested historically (Butt et al. 2005) and the transformation of the landscape with agricultural expansion largely came at the expense of forest habitat. Previous work in the region has showed that the forest bird guild is the one that declines most strongly as amount of agriculture increases (Wilson et al. 2017) and that this response is due primarily to a loss of specific groups including Neotropical migrants, foliage-gleaning insectivores and bark foragers (Endenburg et al. 2019).

Unexpectedly, we found that the richness of shrub-edge birds increases with the amount of agriculture, whether cropland or perennial forages. However, while the direct effect of agriculture on this guild is positive, the indirect effect is negative, such that the total effect is reduced by almost half due to the negative indirect effect on more natural land covers. This overall positive effect suggests that shrub-edge nesting species benefit from agriculture, most likely for feeding in open agricultural land or possibly through a reduction in predator abundance, despite the associated loss of breeding habitats. We also found that agriculture positively influenced the presence of hedgerows, which are frequently used by this guild for breeding and movement and have been shown elsewhere to benefit richness and abundance of shrub-edge species across a range of ecosystems (Hinsley and Bellamy 2000; Benton et al. 2003; Wilson et al. 2017; Montgomery et al. 2020). Thus, overall, shrub-edge bird richness is maximized in agricultural landscapes where fields are interspersed with forest edges and small or linear patches of woody vegetation (see also Fahrig et al. 2015; Wilson et al. 2017; de Zwaan et al. 2022).

Interestingly, we also found that the relationship between the shrub-edge guild and agriculture changed when we focused only on species in long-term decline. Specifically, there was an even greater positive effect of perennial forages and grassland while the previous positive indirect effect of cropland became slightly negative. Declining shrub-edge species also had a stronger relationship with shrubland rather than forest edge and thus one possible explanation for the change in the agricultural effects is that this declining group is affected by loss of shrubland due to cropland expansion, but they otherwise benefit by landscapes where shrubland is embedded in areas of pasture and grassland. Further studies addressing how declining shrub-edge species use agricultural landscapes and the impacts of forage versus cropland agriculture would be useful.

We found an overall positive effect of cropland on open country bird richness; however, the effect of croplands on open country birds is complex. The overall positive effect of cropland was reduced by a third because cropland resulted in a loss of perennial forages and grasslands, which the open country guild most directly benefits from. However, the positive direct effect of cropland on open country birds indicates that this guild benefit directly from cropland as well; this may be due to the use of row crop fields for nesting by some species (e.g. Vesper Sparrow *Pooecetes gramineus*; Jones and Cornely 2020), but many species do not nest in crops and are likely benefiting instead from the grassy edges at the boundaries of crop fields (e.g. Evans et al. 2014). These microhabitats would still appear as a direct effect because they are associated with the crop fields. Alternatively, as suggested for shrub-edge birds, cropland may result in a reduction in predator abundances.

Opposite to our prediction, we found that the overall effect of mean field size on open country bird richness was weak and slightly negative. This result was surprising because many open country birds are thought to benefit from larger fields because of higher nest predation closer to field edges (see predictions in Table 1; Herkert et al. 2003). One explanation for this result may be that open country birds are still more likely to select field edges (e.g., grassy strips) even if those edges include woody vegetation where nest predators may be more common. Another explanation may be that larger fields tend to be more intensively managed than smaller fields and these intensive practices may negatively impact open country birds (Tscharntke et al. 2005; Stanton et al. 2018; Lin and Huang 2019). For example, while larger fields offer more potential habitat, they may also receive greater fertilizer and pesticide inputs and may also be farmed more intensively (e.g. mechanization; Jeliazkov et al. 2016, Martin et al. 2020). This result also suggests that landscape composition (amount of grassland/perennial forages) is a stronger driver of open country bird richness than landscape configuration (mean field size) in our region. This is opposite to results for open country birds elsewhere, such as grassland specialists in the Prairies, where mean field size has a stronger (positive) effect than landscape composition (Lockhart and Koper 2018), although, grassland composition remains important for grassland birds in the Prairies (McDonald and Koper 2022; Pavlacky et al. 2022). We speculate that the amount of grassland may be the limiting factor for open country birds in our region, because most of the region was previously forested with few native grasslands. In such cases, the amount of agriculture may be the key limiting factor. Open country birds therefore occupy most available habitat irrespective of its distance to field edges.

We acknowledge caveats that should be considered with respect to our study. First, because our surveys were conducted along roads there may be biases in the species identified if some species are less likely to select habitats near roads (Harris and Haskell 2007). While this could result in some species in the community being poorly represented in our study it should not affect our examination of direct and indirect effects because this influence would be the same across all of our sites. Second, there may have been other landscape configuration, composition or other agricultural variables that we did not consider that may have influenced species richness of one or more guilds. For example, we did not include specific agricultural practices (e.g. tilling, pesticide usage, timing of cutting, etc.) but these are also known to affect bird diversity and biodiversity in general (Frei et al. 2018; Martin et al. 2020) and therefore could be included in future studies on the impacts of agriculture on biodiversity using structural equation modeling. Third, we only measured guild richness as the biological response because abundance can be difficult to determine with acoustic recording units. However, other measurements such as species diversity or abundance could also be considered in future studies and would allow for a more comprehensive understanding of how other components of biodiversity are affected by the direct and indirect effects of agriculture. Finally, by using guild-level richness as our response variable we are not accounting for turnover of species within guilds following a change in the amount of agriculture. While this does not affect the value of richness as a measure of biodiversity change, it does mean that we may underestimate the effect of agriculture on some species within the guild if other more tolerant species are able to compensate for their loss. Examining direct and indirect effects on individual species of conservation concern is beyond the scope of this analysis but is a potential area of future study.

An understanding of the direct and indirect effects of agriculture on species allows us to make more specific recommendations for conservation. For forest birds, management should focus on retaining existing large forest patches and allowing forest recovery, for example on abandoned agricultural sites. For shrub-edge and open country birds, management could implement policies aimed at shifting the relative amounts of crops vs. perennial forages in favour of the latter. In addition, maximizing forest edge will benefit shrub-edge species. Thus, a bird-friendly agricultural landscape in our region would have forest that is configured to maximize forest edge, and a high proportion of perennial forage within the agricultural portion of the landscape. While our study focused on birds in temperate regions, the approach used here has broad potential to more specifically identify the process by which agriculture impacts species across different taxa and ecosystems.

## Electronic supplementary material

Supplementary table for Tables (DOCX 717 kb)

## Data Availability

The datasets generated and analysed during the current study are available from the corresponding author on reasonable request.
