# Supplementary material for: Direct and indirect effects of agricultural land cover on avian biodiversity in eastern Canada
Source: Biodivers Conserv. 2023 Mar 4;32(4):1403–21. doi: 10.1007/s10531-023-02559-1 (PMC10039827; doi:10.1007/s10531-023-02559-1)
Supplement: Supplementary file 1 — Supplementary table for Tables (DOCX 717 kb) [file 10531_2023_2559_MOESM1_ESM.docx]

SUPPLEMENTAL MATERIAL


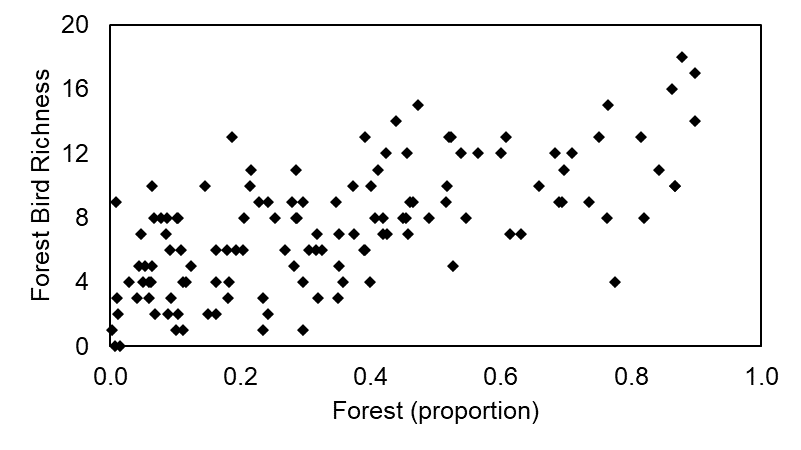


**Fig. S1** Forest bird richness vs. proportion forest in the 1 km^2^ square landscapes surrounding 127 bird survey sites in eastern Ontario, Canada (see Figure 1).


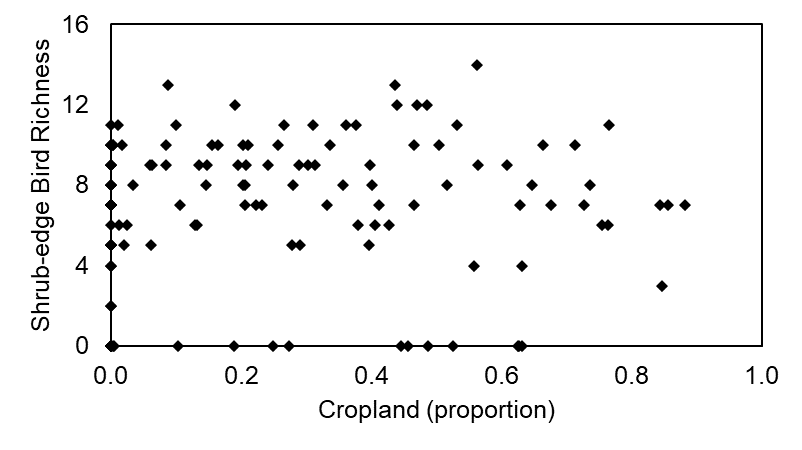


**Fig. S2** Shrub-edge bird richness vs. proportion cropland in the 1 km^2^ square landscapes surrounding 127 bird survey sites in eastern Ontario, Canada (see Figure 1).

*
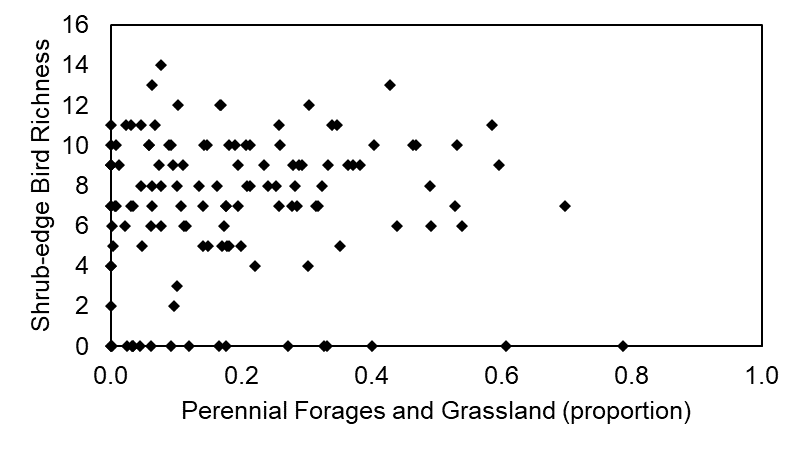
*

**Fig. S3** Shrub-edge bird richness vs. proportion perennial forages and grassland in the 1 km^2^ square landscapes surrounding 127 bird survey sites in eastern Ontario, Canada (see Figure 1).


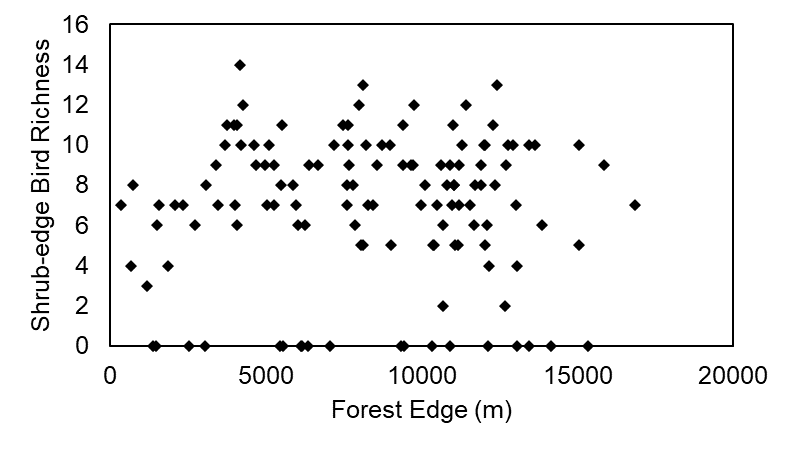


**Fig. S4** Shrub-edge bird richness vs. forest edge (m) in the 1 km^2^ square landscapes surrounding 127 bird survey sites in eastern Ontario, Canada (see Figure 1).


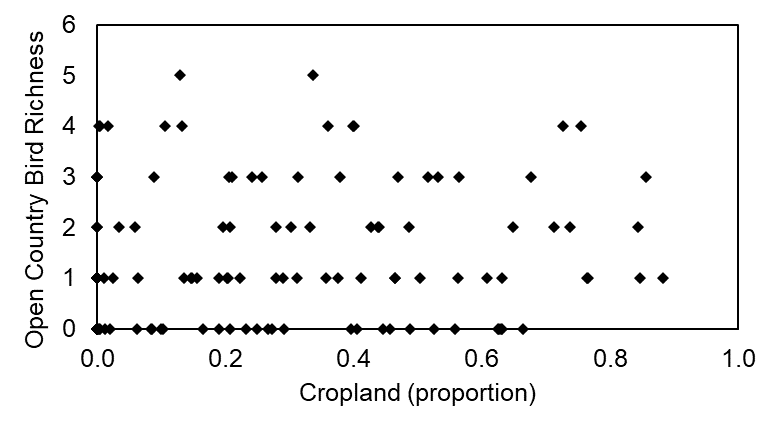


**Fig. S5** Open country bird richness vs. proportion cropland in the 1 km^2^ square landscapes surrounding 127 bird survey sites in eastern Ontario, Canada (see Figure 1).


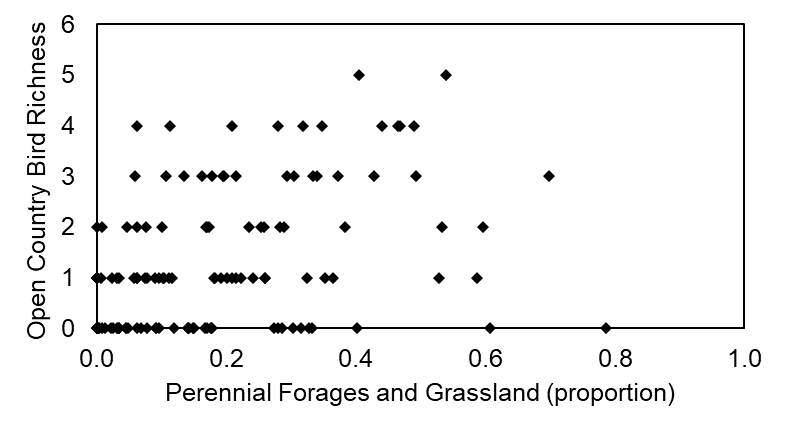


**Fig. S6** Open country bird richness vs. proportion perennial forages and grassland in the 1 km^2^ square landscapes surrounding 127 bird survey sites in eastern Ontario, Canada (see Figure 1).


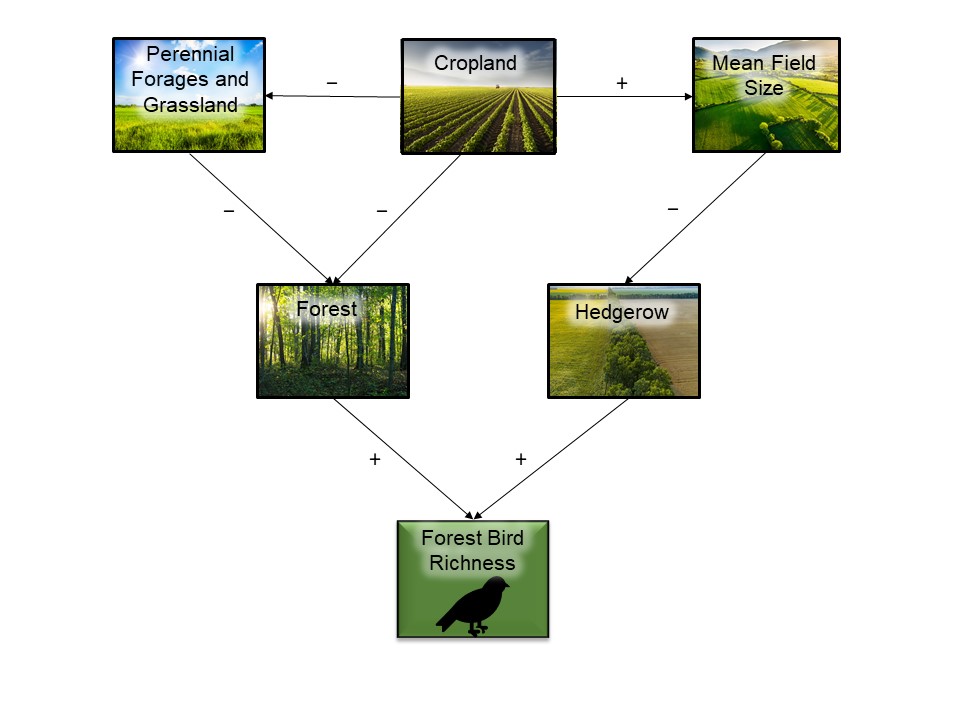


**Fig. S7** Predicted forest bird structural equation modelling (SEM) diagram. Predicted relationships are represented as links originating from the predictor and terminating at the response. The predicted relationship is either positive shown as +, or negative shown as −.


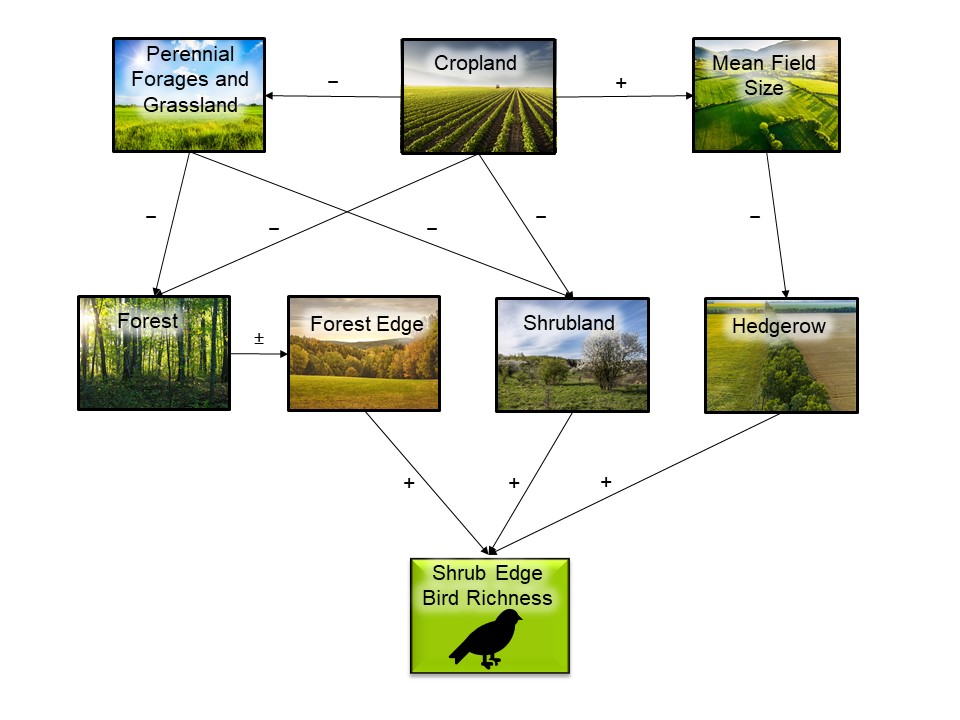


**Fig. S8** Predicted shrub-edge bird structural equation modelling (SEM) diagram. Predicted relationships are represented as links originating from the predictor and terminating at the response. The predicted relationship is positive shown as +, negative shown as −, or peaked non-linear shown as ±.


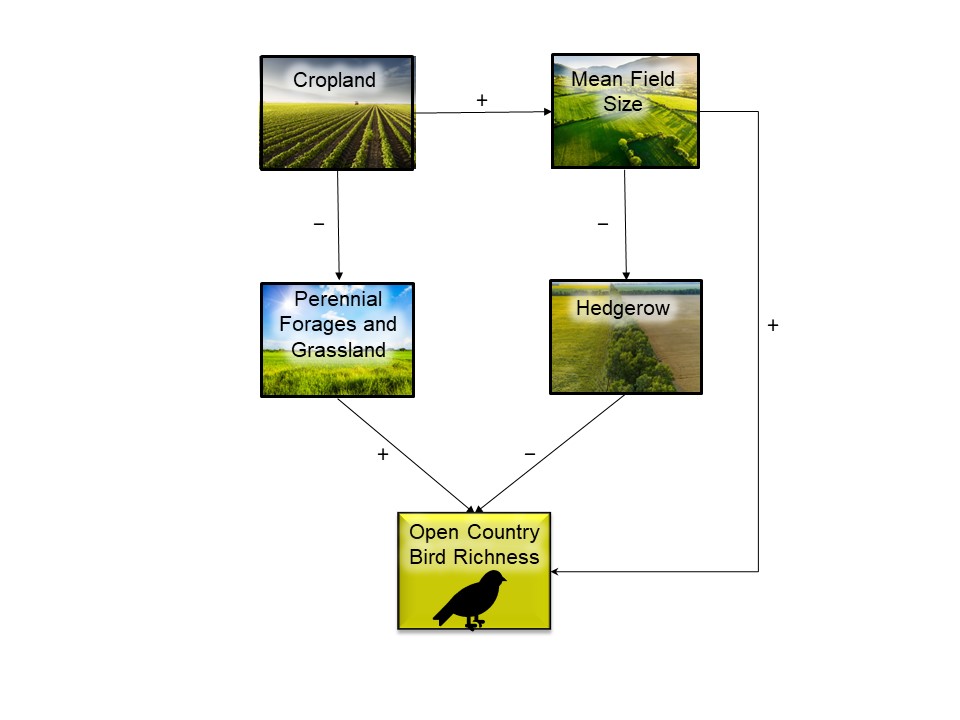


**Fig. S9** Predicted open country bird structural equation modelling (SEM) diagram. Predicted relationships are represented as links originating from the predictor and terminating at the response. The predicted relationship is either positive shown as +, or negative shown as −.


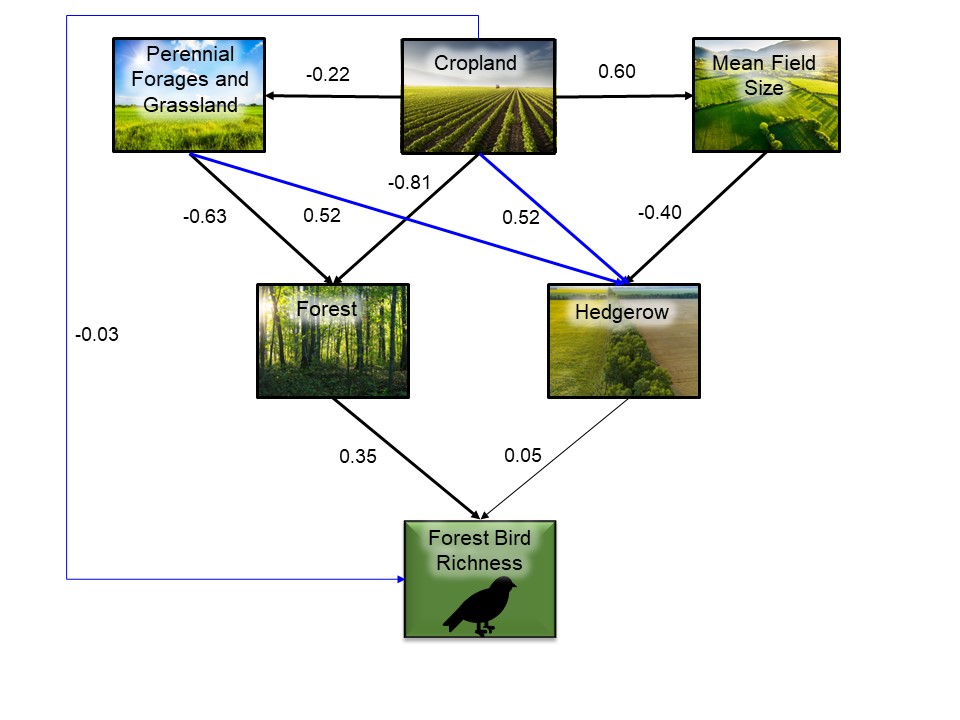


**Fig. S10** Forest bird structural equation modelling (SEM) diagram for declining species with estimates for relationships between agricultural variables, more natural land cover variables, and forest bird richness for declining species. Paths with strong statistical support are represented by thick solid links and paths with weak support are represented by thin solid links. Blue links represent relationships not predicted a priori.


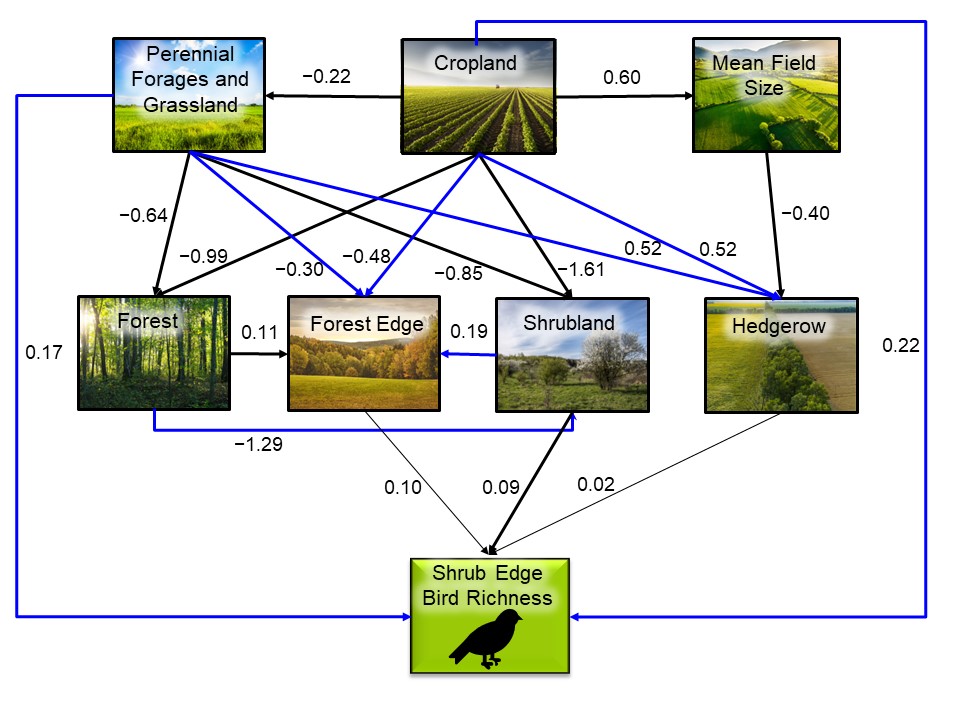


**Fig. S11** Shrub-edge bird structural equation modelling (SEM) diagram for declining species with estimates for relationships between agricultural variables, more natural land cover variables, and shrub-edge bird richness for declining species. Paths with strong statistical support are represented by thick solid links and paths with weak support are represented by thin solid links. Blue links represent relationships not predicted a priori.


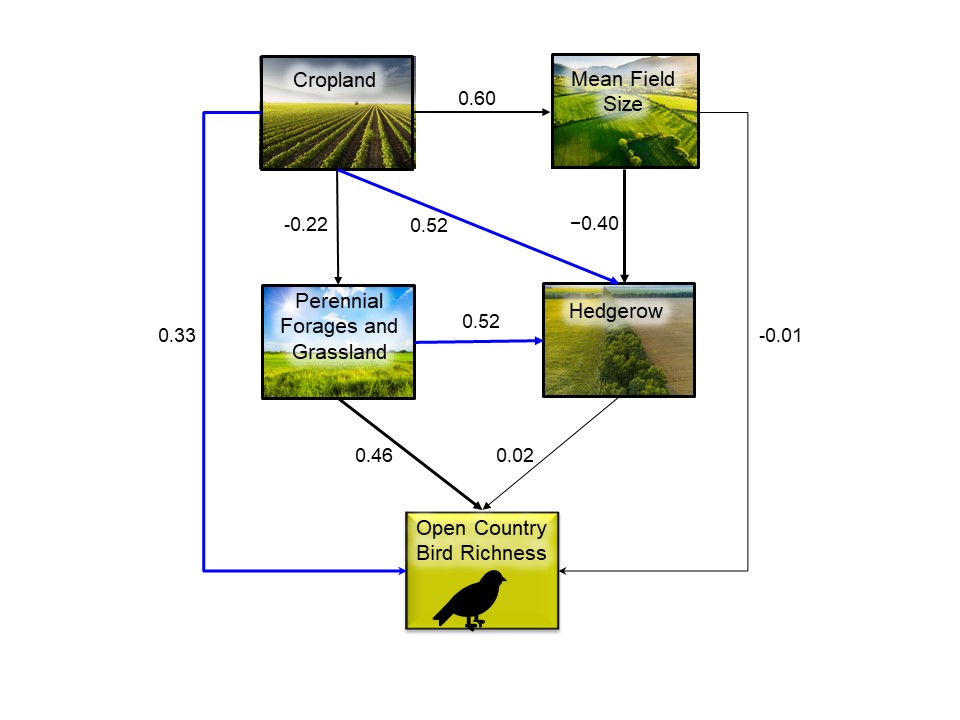


**Fig. S12** Open country bird structural equation modelling (SEM) diagram for declining species with estimates for relationships between agricultural variables, more natural land cover variables, and open country bird richness for declining species. Paths with strong statistical support are represented by thick solid links and paths with weak support are represented by thin solid links. Blue links represent relationships not predicted a priori.

**Table S1** List of bird species identified in the bird surveys including the scientific name, common name, assigned habitat guild (forest, grassland, shrub-edge, and wetland) based on breeding habitat associations provided in the Birds of North America Online, and the number of sites (out of 127 total sites) where each species was identified.

| **Common Name** | **Scientific Name** | **Habitat Guild** | **Sites Identified** |
| --- | --- | --- | --- |
| Alder Flycatcher | *Empidonax alnorum* | Shrub-edge | 23 |
| American Bittern | *Botaurus lentiginosus* | Wetland | 6 |
| American Crow | *Corvus brachyrhynchos* | Shrub-edge | 122 |
| American Goldfinch | *Spinus tristis* | Shrub-edge | 101 |
| American Redstart | *Setophaga ruticilla* | Forest | 27 |
| American Robin | *Turdus migratorius* | Shrub-edge | 125 |
| Baltimore Oriole | *Icterus galbula* | Forest | 15 |
| Barn Swallow | *Hirundo rustica* | Open country | 9 |
| Belted Kingfisher | *Megaceryle alcyon* | Wetland | 2 |
| Black-and-white Warbler | *Mniotilta varia* | Forest | 28 |
| Blackburnian Warbler | *Setophaga fusca* | Forest | 6 |
| Black-capped Chickadee | *Poecile atricapillus* | Forest | 81 |
| Black-crowned Night-Heron | *Nycticorax nycticorax* | Wetland | 1 |
| Black-throated Blue Warbler | *Setophaga caerulescens* | Forest | 1 |
| Black-throated Green Warbler | *Setophaga virens* | Forest | 11 |
| Blue Jay | *Cyanocitta cristata* | Forest | 55 |
| Blue-headed Vireo | *Vireo solitarius* | Forest | 6 |
| Bobolink | *Dolichonyx oryzivorus* | Open country | 29 |
| Broad-winged Hawk | *Buteo platypterus* | Forest | 1 |
| Brown Creeper | *Certhia americana* | Forest | 3 |
| Brown Thrasher | *Toxostoma rufum* | Shrub-edge | 29 |
| Brown-headed Cowbird | *Molothrus ater* | Open country | 18 |
| Canada Goose | *Branta canadensis* | Wetland | 2 |
| Cedar Waxwing | *Bombycilla cedrorum* | Shrub-edge | 48 |
| Chestnut-sided Warbler | *Setophaga pensylvanica* | Shrub-edge | 11 |
| Chipping Sparrow | *Spizella passerina* | Forest | 70 |
| Common Grackle | *Quiscalus quiscula* | Shrub-edge | 44 |
| Common Loon | *Gavia immer* | Wetland | 4 |
| Common Nighthawk | *Chordeiles minor* | Open country | 2 |
| Common Raven | *Corvus corax* | Forest | 26 |
| Common Yellowthroat | *Geothlypis trichas* | Wetland | 97 |
| Dark-eyed Junco | *Junco hyemalis* | Forest | 5 |
| Downy Woodpecker | *Dryobates pubescens* | Forest | 15 |
| Eastern Bluebird | *Sialia sialis* | Open country | 6 |
| Eastern Kingbird | *Tyrannus tyrannus* | Open country | 35 |
| Eastern Meadowlark | *Sturnella magna* | Open country | 20 |
| Eastern Phoebe | *Sayornis phoebe* | Shrub-edge | 43 |
| Eastern Towhee | *Pipilo erythrophthalmus* | Shrub-edge | 2 |
| Eastern Wood Pewee | *Contopus virens* | Forest | 28 |
| European Starling | *Sturnus vulgaris* | Shrub-edge | 26 |
| Field Sparrow | *Spizella pusilla* | Shrub-edge | 9 |
| Golden-crowned Kinglet | *Regulus satrapa* | Forest | 3 |
| Gray Catbird | *Dumetella carolinensis* | Shrub-edge | 30 |
| Great Blue Heron | *Ardea herodias* | Wetland | 3 |
| Great Crested Flycatcher | *Myiarchus crinitus* | Forest | 48 |
| Hairy Woodpecker | *Dryobates villosus* | Forest | 28 |
| Hermit Thrush | *Catharus guttatus* | Forest | 6 |
| Horned Lark | *Eremophila alpestris* | Open country | 1 |
| House Finch | *Haemorhous mexicanus* | Shrub-edge | 4 |
| House Sparrow | *Passer domesticus* | Shrub-edge | 4 |
| House Wren | *Troglodytes aedon* | Shrub-edge | 51 |
| Indigo Bunting | *Passerina cyanea* | Shrub-edge | 1 |
| Killdeer | *Charadrius vociferus* | Open country | 9 |
| Least Flycatcher | *Empidonax minimus* | Forest | 5 |
| Magnolia Warbler | *Setophaga magnolia* | Forest | 6 |
| Mallard | *Anas platyrhynchos* | Wetland | 1 |
| Marsh Wren | *Cistothorus palustris* | Wetland | 2 |
| Mourning Dove | *Zenaida macroura* | Shrub-edge | 99 |
| Mourning Warbler | *Geothlypis philadelphia* | Shrub-edge | 9 |
| Nashville Warbler | *Leiothlypis ruficapilla* | Shrub-edge | 5 |
| Northern Cardinal | *Cardinalis cardinalis* | Shrub-edge | 52 |
| Northern Flicker | *Colaptes auratus* | Forest | 39 |
| Northern Mockingbird | *Mimus polyglottos* | Shrub-edge | 3 |
| Northern Parula | *Setophaga americana* | Forest | 1 |
| Northern Saw-whet Owl | *Aegolius acadicus* | Forest | 1 |
| Northern Waterthrush | *Parkesia noveboracensis* | Forest | 7 |
| Ovenbird | *Seiurus aurocapilla* | Forest | 41 |
| Philadelphia Vireo | *Vireo philadelphicus* | Forest | 3 |
| Pileated Woodpecker | *Dryocopus pileatus* | Forest | 8 |
| Pine Siskin | *Spinus pinus* | Shrub-edge | 4 |
| Pine Warbler | *Setophaga pinus* | Forest | 7 |
| Purple Finch | *Haemorhous purpureus* | Forest | 12 |
| Red-breasted Nuthatch | *Sitta canadensis* | Forest | 13 |
| Red-eyed Vireo | *Vireo olivaceus* | Forest | 79 |
| Red-winged Blackbird | *Agelaius phoeniceus* | Wetland | 93 |
| Ring-billed Gull | *Larus delawarensis* | Wetland | 5 |
| Rose-breasted Grosbeak | *Pheucticus ludovicianus* | Forest | 33 |
| Ruby-crowned Kinglet | *Regulus calendula* | Forest | 1 |
| Ruby-throated Hummingbird | *Archilochus colubris* | Forest | 1 |
| Savannah Sparrow | *Passerculus sandwichensis* | Open country | 44 |
| Scarlet Tanager | *Piranga olivacea* | Forest | 7 |
| Song Sparrow | *Melospiza melodia* | Shrub-edge | 115 |
| Spotted Sandpiper | *Actitis macularius* | Wetland | 2 |
| Swamp Sparrow | *Melospiza georgiana* | Wetland | 13 |
| Upland Sandpiper | *Bartramia longicauda* | Open country | 2 |
| Veery | *Catharus fuscescens* | Forest | 38 |
| Vesper Sparrow | *Pooecetes gramineus* | Open country | 16 |
| Warbling Vireo | *Vireo gilvus* | Forest | 46 |
| White-breasted Nuthatch | *Sitta carolinensis* | Forest | 25 |
| White-throated Sparrow | *Zonotrichia albicollis* | Forest | 32 |
| Wild Turkey | *Meleagris gallopavo* | Forest | 14 |
| Wilson's Snipe | *Gallinago delicata* | Wetland | 2 |
| Wilson's Warbler | *Cardellina pusilla* | Shrub-edge | 1 |
| Winter Wren | *Troglodytes hiemalis* | Forest | 3 |
| Wood Thrush | *Hylocichla mustelina* | Forest | 20 |
| Yellow Warbler | *Setophaga petechia* | Shrub-edge | 48 |
| Yellow-bellied Sapsucker | *Sphyrapicus varius* | Forest | 25 |
| Yellow-billed Cuckoo | *Coccyzus americanus* | Forest | 4 |
| Yellow-rumped Warbler | *Setophaga coronata* | Forest | 4 |
| Yellow-throated Vireo | *Vireo flavifrons* | Forest | 1 |

**Table S2** Bird richness by site for each bird guild (forest, open country, shrub-edge and wetland birds) and for all birds (total richness). Includes the average bird richness, and maximum bird richness.

| **Site ID** | **Forest Bird Richness** | **Open Country Bird Richness** | **Shrub-Edge Bird Richness** | **Wetland Bird Richness** | **Total Richness** |
| --- | --- | --- | --- | --- | --- |
| A102 | 8 | 4 | 6 | 2 | 20 |
| A103 | 11 | 1 | 9 | 3 | 24 |
| A104 | 4 | 2 | 8 | 3 | 17 |
| A108 | 9 | 0 | 11 | 2 | 22 |
| A109 | 8 | 3 | 13 | 3 | 27 |
| A110 | 5 | 5 | 10 | 1 | 21 |
| A12 | 14 | 2 | 8 | 2 | 26 |
| A121 | 2 | 5 | 6 | 1 | 14 |
| A125 | 6 | 2 | 8 | 3 | 19 |
| A134 | 8 | 1 | 10 | 3 | 22 |
| A137 | 8 | 3 | 12 | 3 | 26 |
| A144 | 4 | 3 | 7 | 3 | 17 |
| A149 | 2 | 2 | 9 | 1 | 14 |
| A150 | 8 | 1 | 10 | 2 | 21 |
| A152A | 4 | 0 | 0 | 0 | 4 |
| A157 | 2 | 4 | 8 | 1 | 15 |
| A158 | 3 | 3 | 7 | 3 | 16 |
| A159 | 13 | 3 | 9 | 2 | 27 |
| A169 | 16 | 0 | 7 | 2 | 25 |
| A177 | 0 | 0 | 4 | 2 | 6 |
| A186 | 4 | 1 | 4 | 0 | 9 |
| A187 | 12 | 3 | 9 | 2 | 26 |
| A193 | 9 | 3 | 8 | 3 | 23 |
| A197A | 14 | 0 | 0 | 0 | 14 |
| A202A | 13 | 0 | 0 | 0 | 13 |
| A204 | 10 | 0 | 6 | 0 | 16 |
| A206A | 4 | 0 | 0 | 0 | 4 |
| A211 | 12 | 0 | 7 | 2 | 21 |
| A212 | 13 | 0 | 2 | 0 | 15 |
| A218 | 12 | 0 | 5 | 2 | 19 |
| A29 | 8 | 2 | 13 | 3 | 26 |
| A32A | 11 | 0 | 0 | 0 | 11 |
| A39 | 5 | 2 | 10 | 2 | 19 |
| A42 | 6 | 2 | 9 | 2 | 19 |
| A43 | 5 | 1 | 8 | 2 | 16 |
| A47 | 4 | 4 | 6 | 2 | 16 |
| A48 | 8 | 1 | 8 | 2 | 19 |
| A49 | 4 | 1 | 14 | 2 | 21 |
| A5 | 10 | 0 | 6 | 1 | 17 |
| A54 | 7 | 0 | 10 | 2 | 19 |
| A56 | 10 | 1 | 10 | 3 | 24 |
| A62A | 3 | 0 | 0 | 0 | 3 |
| A69 | 9 | 4 | 10 | 2 | 25 |
| A7 | 7 | 1 | 10 | 3 | 21 |
| A72 | 2 | 2 | 7 | 1 | 12 |
| A73 | 12 | 1 | 8 | 3 | 24 |
| A74 | 13 | 1 | 8 | 1 | 23 |
| A75 | 4 | 1 | 11 | 2 | 18 |
| A76 | 10 | 0 | 7 | 1 | 18 |
| A78 | 6 | 1 | 7 | 3 | 17 |
| A80 | 2 | 4 | 11 | 2 | 19 |
| A85 | 8 | 1 | 7 | 2 | 18 |
| A86A | 6 | 0 | 0 | 0 | 6 |
| A89 | 3 | 2 | 10 | 3 | 18 |
| A90 | 7 | 1 | 7 | 3 | 18 |
| B10 | 8 | 0 | 7 | 2 | 17 |
| B100 | 18 | 0 | 5 | 1 | 24 |
| B104 | 7 | 0 | 10 | 2 | 19 |
| B106A | 8 | 0 | 0 | 0 | 8 |
| B107 | 7 | 0 | 6 | 1 | 14 |
| B108 | 8 | 1 | 9 | 2 | 20 |
| B109 | 0 | 1 | 3 | 3 | 7 |
| B110 | 1 | 3 | 7 | 1 | 12 |
| B111 | 5 | 1 | 8 | 2 | 16 |
| B114 | 10 | 3 | 11 | 2 | 26 |
| B116 | 6 | 2 | 12 | 3 | 23 |
| B117 | 6 | 1 | 7 | 1 | 15 |
| B118 | 12 | 1 | 12 | 2 | 27 |
| B11A | 1 | 0 | 0 | 0 | 1 |
| B12 | 11 | 0 | 5 | 1 | 17 |
| B120 | 4 | 1 | 6 | 2 | 13 |
| B124 | 5 | 3 | 7 | 2 | 17 |
| B127 | 6 | 3 | 9 | 2 | 20 |
| B13 | 3 | 2 | 6 | 2 | 13 |
| B17 | 9 | 1 | 5 | 0 | 15 |
| B1A | 10 | 0 | 0 | 0 | 10 |
| B2 | 9 | 0 | 5 | 2 | 16 |
| B26 | 9 | 4 | 10 | 2 | 25 |
| B28 | 8 | 1 | 9 | 2 | 20 |
| B35 | 13 | 0 | 5 | 1 | 19 |
| B37 | 7 | 0 | 10 | 2 | 19 |
| B38A | 6 | 0 | 0 | 0 | 6 |
| B39 | 3 | 4 | 10 | 2 | 19 |
| B4 | 2 | 2 | 8 | 3 | 15 |
| B40 | 10 | 1 | 9 | 2 | 22 |
| B41 | 6 | 1 | 7 | 3 | 17 |
| B42 | 6 | 4 | 9 | 2 | 21 |
| B44 | 6 | 0 | 7 | 1 | 14 |
| B45 | 10 | 1 | 10 | 2 | 23 |
| B46 | 5 | 2 | 7 | 2 | 16 |
| B48 | 8 | 3 | 10 | 3 | 24 |
| B49 | 15 | 0 | 11 | 1 | 27 |
| B50 | 5 | 3 | 6 | 1 | 15 |
| B51 | 10 | 1 | 11 | 4 | 26 |
| B52A | 9 | 0 | 0 | 0 | 9 |
| B53 | 1 | 3 | 8 | 1 | 13 |
| B56 | 3 | 3 | 9 | 2 | 17 |
| B58A | 7 | 0 | 0 | 0 | 7 |
| B59 | 2 | 1 | 11 | 1 | 15 |
| B5A | 13 | 0 | 0 | 0 | 13 |
| B6 | 13 | 0 | 5 | 0 | 18 |
| B61 | 3 | 4 | 7 | 2 | 16 |
| B64A | 7 | 0 | 0 | 0 | 7 |
| B68 | 1 | 1 | 6 | 2 | 10 |
| B69 | 11 | 0 | 9 | 1 | 21 |
| B71 | 12 | 1 | 5 | 2 | 20 |
| B72A | 4 | 0 | 0 | 0 | 4 |
| B73 | 3 | 2 | 8 | 1 | 14 |
| B74 | 8 | 1 | 9 | 0 | 18 |
| B79 | 17 | 0 | 9 | 2 | 28 |
| B81 | 4 | 4 | 7 | 3 | 18 |
| B82 | 9 | 2 | 12 | 2 | 25 |
| B83 | 4 | 3 | 10 | 2 | 19 |
| B84 | 9 | 2 | 9 | 2 | 22 |
| B85 | 15 | 1 | 11 | 1 | 28 |
| B86 | 11 | 2 | 9 | 2 | 24 |
| B87 | 9 | 0 | 11 | 2 | 22 |
| B9 | 6 | 0 | 7 | 0 | 13 |
| B93A | 8 | 0 | 0 | 0 | 8 |
| C1A | 7 | 0 | 0 | 0 | 7 |
| C2A | 9 | 0 | 0 | 0 | 9 |
| C3 | 12 | 0 | 4 | 0 | 16 |
| C4 | 8 | 0 | 2 | 0 | 10 |
| C5 | 8 | 1 | 4 | 0 | 13 |
| C6 | 9 | 1 | 9 | 2 | 21 |
| C7 | 7 | 1 | 10 | 2 | 20 |
| C9 | 1 | 1 | 5 | 2 | 9 |
| **AVERAGE** | **7** | **1** | **7** | **2** | **17** |
| **MAX** | **18** | **5** | **14** | **4** | **28** |


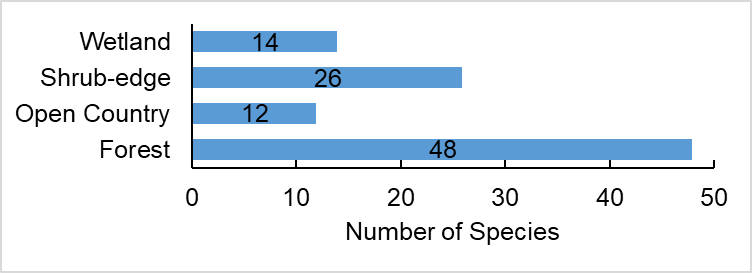


**Fig. S13** Total number of species identified, grouped by bird guild (wetland, shrub-edge, open country and forest birds).

**Table S3** Summary statistics of landscape variables (proportion forest, cropland, shrubland, wetland, hedgerows, perennial forages and grassland, and mean field size in hectares and total forest edge in meters) measured in 1 km^2^ landscapes around each bird survey site, including the average and the range of each variable. Note: mean field size range starts at 0 as 7 sites had no agricultural fields.

| **Landscape Variable** | **Average** | **Range** |
| --- | --- | --- |
| Proportion Forest | 0.346 | 0.03 - 0.9 |
| Proportion Cropland | 0.263 | 0 - 0.88 |
| Proportion Shrubland | 0.068 | 0.001 - 0.41 |
| Proportion Wetland | 0.031 | 0 - 0.3 |
| Proportion Hedgerows | 0.014 | 0 - 0.07 |
| Proportion Perennial Forages and Grassland | 0.192 | 0 - 0.79 |
| Mean Field Size (ha) | 12.764 | 0 - 88.2 |
| Forest edge (m) | 8344.9 | 352.4 - 16839.3 |

**Table S4** Long-term (1970 to 2019) population trends in Canada for all bird species from the North American Breeding Bird Survey. SAR = Species-At-Risk (0 = not at risk, 1 = Species-At-Risk). Trend <0% indicates if long-term trends are declining (0 = not declining, 1 = declining) based on mean trends and 95% credible intervals below 0. LCI = lower 95% credible interval, UCI = upper 95% credible interval.

| **Common Name** | **Sites** | **SAR** | **Trends < 0%** | **Trend** | **LCI** | **UCI** |
| --- | --- | --- | --- | --- | --- | --- |
| Alder Flycatcher | 23 | 0 | 0 | 1.31 | -34.8 | 40.8 |
| American Bittern | 6 | 0 | 0 | -21.6 | -51.6 | 31 |
| American Crow | 122 | 0 | 1 | -33.2 | -41.5 | -24.7 |
| American Goldfinch | 101 | 0 | 1 | -32 | -42.7 | -21.2 |
| American Redstart | 27 | 0 | 0 | 6.55 | -26.6 | 35.9 |
| American Robin | 125 | 0 | 0 | 4.46 | -20.7 | 27.7 |
| Baltimore Oriole | 15 | 0 | 1 | -52.1 | -62.5 | -40.7 |
| Barn Swallow | 9 | 1 | 1 | -68.6 | -73.3 | -63.8 |
| Belted Kingfisher | 2 | 0 | 1 | -37.4 | -51.7 | -19.3 |
| Black-and-white Warbler | 28 | 0 | 0 | 3.55 | -32 | 40.4 |
| Blackburnian Warbler | 6 | 0 | 0 | 20.6 | -18.9 | 78.8 |
| Black-capped Chickadee | 81 | 0 | 0 | 51 | 25.2 | 81.1 |
| Black-crowned Night-Heron | 1 | 0 | 0 | 0.732 | -64.5 | 185 |
| Black-throated Blue Warbler | 1 | 0 | 0 | 145 | 65 | 260 |
| Black-throated Green Warbler | 11 | 0 | 0 | -4.5 | -37.7 | 32.3 |
| Blue Jay | 55 | 0 | 0 | 65.5 | 44.6 | 89.9 |
| Blue-headed Vireo | 6 | 0 | 0 | 422 | 234 | 652 |
| Bobolink | 29 | 1 | 1 | -72.9 | -77.4 | -67.5 |
| Broad-winged Hawk | 1 | 0 | 0 | 111 | 58.4 | 184 |
| Brown Creeper | 3 | 0 | 0 | 134 | 49.1 | 272 |
| Brown Thrasher | 29 | 0 | 1 | -38.4 | -48.6 | -26.2 |
| Brown-headed Cowbird | 18 | 0 | 1 | -42.1 | -50.3 | -32.6 |
| Canada Goose | 2 | 0 | 0 | 12800 | 689 | 63600 |
| Cedar Waxwing | 48 | 0 | 0 | 5.79 | -44.7 | 60.1 |
| Chestnut-sided Warbler | 11 | 0 | 1 | -42.5 | -57.6 | -24.2 |
| Chipping Sparrow | 70 | 0 | 1 | -48 | -78.4 | -22.2 |
| Common Grackle | 44 | 0 | 0 | -9.12 | -25.7 | 10.3 |
| Common Loon | 4 | 0 | 0 | 81.6 | 17.9 | 168 |
| Common Nighthawk | 2 | 0 | 1 | -61 | -78.1 | -34.2 |
| Common Raven | 26 | 0 | 0 | 205 | 65.8 | 357 |
| Common Yellowthroat | 97 | 0 | 1 | -17.3 | -33.4 | -1.64 |
| Dark-eyed Junco | 5 | 0 | 0 | -15.8 | -62.6 | 61.7 |
| Downy Woodpecker | 15 | 0 | 0 | 15.1 | -3.46 | 36.6 |
| Eastern Bluebird | 6 | 0 | 0 | 158 | 76.7 | 272 |
| Eastern Kingbird | 35 | 0 | 1 | -38.7 | -48.3 | -27.7 |
| Eastern Meadowlark | 20 | 1 | 1 | -88 | -90.1 | -85.3 |
| Eastern Phoebe | 43 | 0 | 0 | -29 | -56 | 10.6 |
| Eastern Towhee | 2 | 0 | 1 | -41.9 | -61.4 | -12.5 |
| Eastern Wood-Pewee | 28 | 1 | 1 | -63 | -72.3 | -54.8 |
| European Starling | 26 | 0 | 1 | -70.3 | -76 | -64.7 |
| Field Sparrow | 9 | 0 | 1 | -45.2 | -59.8 | -26.3 |
| Golden-crowned Kinglet | 3 | 0 | 0 | 23.8 | -19.3 | 90 |
| Gray Catbird | 30 | 0 | 0 | 0.379 | -13.9 | 16.3 |
| Great Blue Heron | 3 | 0 | 0 | -13.5 | -38.3 | 18.3 |
| Great Crested Flycatcher | 48 | 0 | 1 | -27.4 | -38.8 | -14.3 |
| Hairy Woodpecker | 28 | 0 | 0 | 82 | 45.5 | 127 |
| Hermit Thrush | 6 | 0 | 0 | -34.4 | -80.4 | 57.7 |
| Horned Lark | 1 | 0 | 1 | -89.1 | -91.5 | -86.4 |
| House Finch | 4 | 0 | 0 | 128 | -5.94 | 485 |
| House Sparrow | 4 | 0 | 1 | -84.7 | -87.1 | -81.8 |
| House Wren | 51 | 0 | 0 | 35.8 | 15.8 | 59.7 |
| Indigo Bunting | 1 | 0 | 0 | 30.2 | 4.7 | 60.5 |
| Killdeer | 9 | 0 | 1 | -70.9 | -75.6 | -65.8 |
| Least Flycatcher | 5 | 0 | 1 | -46.8 | -66.9 | -30.5 |
| Magnolia Warbler | 6 | 0 | 0 | 55.3 | 6.74 | 112 |
| Mallard | 1 | 0 | 0 | -9.58 | -40.8 | 29.4 |
| Marsh Wren | 2 | 0 | 0 | 483 | 154 | 1170 |
| Mourning Dove | 99 | 0 | 0 | 82.6 | 60.3 | 108 |
| Mourning Warbler | 9 | 0 | 1 | -54.1 | -67.4 | -36 |
| Nashville Warbler | 5 | 0 | 0 | -16 | -42.4 | 18.5 |
| Northern Cardinal | 52 | 0 | 0 | 489 | 374 | 639 |
| Northern Flicker | 39 | 0 | 1 | -42.5 | -57 | -27.6 |
| Northern Mockingbird | 3 | 0 | 0 | -15.6 | -64.2 | 83.8 |
| Northern Parula | 1 | 0 | 0 | 328 | 209 | 442 |
| Northern Saw-whet Owl | 1 | 0 | 0 | -23.5 | -81.4 | 77 |
| Northern Waterthrush | 7 | 0 | 0 | 28.8 | -40.7 | 156 |
| Ovenbird | 41 | 0 | 0 | 19.2 | -7.42 | 45.2 |
| Philadelphia Vireo | 3 | 0 | 0 | 224 | 44.9 | 594 |
| Pileated Woodpecker | 8 | 0 | 0 | 309 | 201 | 450 |
| Pine Siskin | 4 | 0 | 1 | -62.9 | -77.1 | -40.9 |
| Pine Warbler | 7 | 0 | 0 | 488 | 176 | 999 |
| Purple Finch | 12 | 0 | 0 | -25.7 | -45.5 | 1.49 |
| Red-breasted Nuthatch | 13 | 0 | 0 | 181 | 117 | 268 |
| Red-eyed Vireo | 79 | 0 | 0 | 40.6 | 2.86 | 67.8 |
| Red-winged Blackbird | 93 | 0 | 0 | -2.97 | -17.6 | 10.3 |
| Ring-billed Gull | 5 | 0 | 0 | 301 | 80.3 | 869 |
| Rose-breasted Grosbeak | 33 | 0 | 1 | -20.5 | -39.5 | -1.19 |
| Ruby-crowned Kinglet | 1 | 0 | 0 | 172 | 21.5 | 374 |
| Ruby-throated Hummingbird | 1 | 0 | 0 | 68.5 | 30.6 | 118 |
| Savannah Sparrow | 44 | 0 | 1 | -37.6 | -68.7 | -2.25 |
| Scarlet Tanager | 7 | 0 | 0 | -20.6 | -42.3 | 5.53 |
| Song Sparrow | 115 | 0 | 1 | -39.7 | -47.9 | -31.7 |
| Spotted Sandpiper | 2 | 0 | 1 | -62.9 | -81.2 | -37.7 |
| Swamp Sparrow | 13 | 0 | 0 | 20.5 | -37.7 | 115 |
| Upland Sandpiper | 2 | 0 | 0 | 56 | -18.6 | 148 |
| Veery | 38 | 0 | 1 | -23.5 | -35.8 | -8.59 |
| Vesper Sparrow | 16 | 0 | 1 | -47.4 | -60.8 | -34.4 |
| Warbling Vireo | 46 | 0 | 0 | 52.6 | 14.9 | 95.9 |
| White-breasted Nuthatch | 25 | 0 | 0 | 147 | 81.3 | 238 |
| White-throated Sparrow | 32 | 0 | 1 | -34 | -59.9 | -5.71 |
| Wild Turkey | 14 | 0 | 0 | 125000 | 17100 | 744000 |
| Wilson's Snipe | 2 | 0 | 0 | 86 | -29.3 | 244 |
| Wilson's Warbler | 1 | 0 | 0 | -9.09 | -53.3 | 69.3 |
| Winter Wren | 3 | 0 | 0 | 8.36 | -32.1 | 63.7 |
| Wood Thrush | 20 | 1 | 1 | -71.2 | -77.8 | -62.8 |
| Yellow Warbler | 48 | 0 | 0 | -15.5 | -49 | 16.2 |
| Yellow-bellied Sapsucker | 25 | 0 | 0 | 20.4 | -35 | 79.8 |
| Yellow-billed Cuckoo | 4 | 0 | 0 | 0.157 | -35.3 | 59.1 |
| Yellow-rumped Warbler | 4 | 0 | 0 | 27.6 | -49.4 | 133 |
| Yellow-throated Vireo | 1 | 0 | 0 | 110 | 16.8 | 264 |

**Table S5** Pearson correlation matrix for the landscape variables measured within 1 km^2^ landscapes surrounding each bird survey site. Correlations > 0.5 or < -0.5 are highlighted in bold. MFS = mean field size, Edge = forest edge, Forages Grassland = proportion perennial forages and grassland, and HR = proportion hedgerows.

|  | **MFS** | **Edge** | **Forest** | **Shrubland** | **Cropland** | **Forages Grassland** | **HR** |
| --- | --- | --- | --- | --- | --- | --- | --- |
| **MFS** | 1 |  |  |  |  |  |  |
| **Edge** | **-0.52** | 1 |  |  |  |  |  |
| **Forest** | -0.39 | **0.72** | 1 |  |  |  |  |
| **Shrubland** | -0.36 | **0.60** | 0.17 | 1 |  |  |  |
| **Cropland** | **0.60** | **-0.74** | **-0.67** | **-0.55** | 1 |  |  |
| **Forages Grassland** | -0.06 | -0.24 | -0.45 | 0.09 | -0.22 | 1 |  |
| **HR** | -0.12 | -0.31 | -0.50 | -0.03 | 0.17 | 0.42 | 1 |

**Table S6** Equations used to calculate the indirect effect of agriculture variables (cropland, perennial forages and grassland, and mean field size) on richness of bird guilds (forest, open country, and shrub-edge bird richness) from links in Figure 3. Indirect effects were derived from the sum of all indirect pathways leading to bird richness from the agricultural variable through a habitat variable, where each pathway is the product of the estimates for all relationships (i.e. links) in the pathway.

| **Guild** | **Agriculture Variable** | **Indirect Pathways (number)** | **Indirect Effect Equation** | **Indirect Effect** |
| --- | --- | --- | --- | --- |
| Forest birds | Cropland | 5 | (-0.81*0.32)+(0.52*0.05)+(0.6*-0.4*0.05)+(-0.22*-0.63*0.32)+(-0.22*0.52*0.05) | -0.207 |
| Shrub-Edge birds | Cropland | 15 | (0.6*-0.4*0.04)+(0.52*0.04)+(-1.61*0.05)+(-0.48*0.17)+(-0.99*0.11*0.17)+(-0.99*-1.29*0.05)+(-0.99*-1.29*0.19*0.17)+(-0.22*0.1)+(-0.22*-0.64*0.11*0.17)+(-0.22*-0.64*-1.29*0.05)+(-0.22*-0.64*-1.29*0.19+0.17)+(-0.22*-0.3*0.17)+(-0.22*-0.85*0.05)+(-0.22*-0.85*0.19*0.17)+(-0.22*0.52*0.04) | 0.065 |
| Shrub-Edge birds | Perennial Forages and Grassland | 7 | (-0.64*0.11*0.17)+(-0.64*-1.29*0.05)+(-0.64*-1.29*0.19*0.17)+(-0.3*0.17)+(-0.85*0.05)+(-0.85*0.19*0.17)+(0.52*0.04) | -0.044 |
| Open country birds | Cropland | 5 | (0.52*0.01)+(0.6*-0.4*0.01)+(0.6*-0.004)+(-0.22*0.52*0.01)+(-0.22*0.45) | -0.100 |
| Open country birds | Perennial Forages and Grassland | 1 | (0.52*0.01) | 0.005 |
| Open country birds | Mean Field Size | 1 | (-0.4*0.01) | -0.004 |

**Table S7** Equations used to calculate the indirect effect of agriculture variables (cropland, perennial forages and grassland, and mean field size) on richness of bird guilds (forest, open country, and shrub-edge bird richness) for declining species. Indirect effects were derived from the sum of all indirect pathways leading to bird richness from the agricultural variable through a habitat variable, where each pathway is the product of the estimates for all relationships (i.e. links) in the pathway.

| **Guild** | **Agriculture Variable** | **Indirect Pathways (number)** | **Indirect Effect Equation** | **Indirect Effect** |
| --- | --- | --- | --- | --- |
| Forest birds | Cropland | 5 | (-0.81*0.35)+(0.52*0.05)+(0.6*-0.4*0.05)+(-0.22*-0.63*0.35)+(-0.22*0.52*0.05) | -0.227 |
| Shrub-Edge birds | Cropland | 15 | (0.6*-0.4*0.02)+(0.52*0.02)+(-1.61*0.09)+(-0.48*0.10)+(-0.99*0.11*0.10)+(-0.99*-1.29*0.09)+(-0.99*-1.29*0.19*0.10)+(-0.22*0.17)+(-0.22*-0.64*0.11*0.10)+(-0.22*-0.64*-1.29*0.09)+(-0.22*-0.64*-1.29*0.19+0.10)+(-0.22*-0.3*0.10)+(-0.22*-0.85*0.09)+(-0.22*-0.85*0.19*0.10)+(-0.22*0.52*0.02) | -0.021 |
| Shrub-Edge birds | Perennial Forages and Grassland | 7 | (-0.64*0.11*0.10)+(-0.64*-1.29*0.09)+(-0.64*-1.29*0.19*0.10)+(-0.3*0.10)+(-0.85*0.09)+(-0.85*0.19*0.10)+(0.52*0.02) | -0.029 |
| Open country birds | Cropland | 5 | (0.52*0.02)+(0.6*-0.4*0.02)+(0.6*-0.01)+(-0.22*0.52*0.02)+(-0.22*0.46) | -0.104 |
| Open country birds | Perennial Forages and Grassland | 1 | (0.52*0.02) | 0.010 |
| Open country birds | Mean Field Size | 1 | (-0.4*0.02) | -0.008 |

**Table S8** Summary of the direct, indirect, and total effects of agriculture variables (cropland, perennial forages and grassland, and mean field size) on bird richness (forest, shrub-edge and open country bird richness) for declining species. Direct effects are derived from the direct relationship (i.e. link) between the agricultural variable and bird richness. Indirect effects were derived from the sum of all indirect pathways leading to bird richness from the agricultural variable, where each pathway is the product of the estimates for all relationships (i.e. link) in the pathway. The proportional change to the total effect is relative to the direct effect, where - represents negative and + represents positive.

| **Bird Richness Guild** | **Agriculture Variable** | **Indirect Pathways (number)** | **Direct Effect** | **Indirect Effect** | **Total Effect** | **Change in total effect for declining species^+^** |
| --- | --- | --- | --- | --- | --- | --- |
| Forest | Cropland | 5 | -0.03 | -0.227 | -0.257 | -0.010 |
| Shrub-Edge | Cropland | 15 | 0.22 | -0.021 | 0.199 | -0.066 |
| Shrub-Edge | Perennial Forages and Grassland | 7 | 0.17 | -0.029 | 0.141 | +0.085 |
| Open Country | Cropland | 5 | 0.33 | -0.104 | 0.226 | -0.014 |
| Open Country | Mean Field Size | 1 | -0.01 | -0.008 | -0.018 | -0.010 |

^+^ Refers to the change relative to the total effect estimate for all species

**Fig. S14** The proportion of agriculture (cropland and perennial forages and grassland) in the local landscape measured within a 150m radius around each site vs. the proportion of agriculture measured in 1 km^2^ landscapes surrounding each bird survey site.

**Fig. S15** The proportion of forest in the local landscape measured within a 150m radius around each site vs. the proportion of forest measured in 1 km^2^ landscapes surrounding each bird survey site.

**Table S9** All conditionally independent pairs of variables from the forest bird SEM diagram (Figure 2), structured as independence claims, implied by the path model of the predicted relationships between landscape predictors and forest bird richness, and the associated models constructed to test the independence claims. Also shown are the null probabilities (p-values) used to calculate Fisher’s C statistic to test the correlational structure of the full path model. The independence claim notation identifies the tested pair of variables in parentheses, followed by the variables that were statistically controlled for while testing the independence between the pair, in curled brackets.

| **Independence Claim** | **Model^a^** | **p-value** |
| --- | --- | --- |
| (MFS, ForageGrass)\|{Crop} | MFS ~ Crop + ForageGrass | 0.30058 |
| (ForestRich, ForageGrass)\|{Crop, Forest} | ForestRich ~ Crop + Forest + ForageGrass | 0.486681 |
| (Forest, MFS)\|{Crop, ForageGrass} | Forest ~ Crop + ForageGrass + MFS | 0.0485 |
| (ForestRich, MFS)\|{Crop, Forest, HR} | ForestRich ~ Crop + Forest + HR + MFS | 0.763 |
| (Forest, HR)\|{Crop, ForageGrass, MFS} | Forest ~ Crop + ForageGrass + MFS + HR | 0.0151 |

*Notes:* MFS = mean field size, ForageGrass = perennial forages and grassland, Crop = cropland, ForestRich = forest bird richness, HR = Hedgerow. ^a^ Relationships with the ForestRich response variable were tested using generalized linear models with a Poisson distribution. Relationships between all other variables were tested using linear models.

**Table S10** All conditionally independent pairs of variables from the open country bird SEM diagram (Figure 4), structured as independence claims, implied by the path model of the predicted relationships between landscape predictors and open country bird richness, and the associated models constructed to test the independence claims. Also shown are the null probabilities (p-values) used to calculate Fisher’s C statistic to test the correlational structure of the full path model. The independence claim notation identifies the tested pair of variables in parentheses, followed by the variables that were statistically controlled for while testing the independence between the pair, in curled brackets.

| **Independence Claim** | **Model^a^** | **p-value** |
| --- | --- | --- |
| (MFS, ForageGrass)\|{Crop} | MFS ~ Crop + ForageGrass | 0.30058 |

*Notes:* MFS = mean field size, ForageGrass = perennial forages and grassland, Crop = cropland, ^a^ Relationships were tested using linear models.

**Table S11** All conditionally independent pairs of variables from the shrub edge bird SEM diagram (Figure 3), structured as independence claims, implied by the path model of the predicted relationships between landscape predictors and shrub-edge bird richness, and the associated models constructed to test the independence claims. Also shown are the null probabilities (p-values) used to calculate Fisher’s C statistic to test the correlational structure of the full path model. The independence claim notation identifies the tested pair of variables in parentheses, followed by the variables that were statistically controlled for while testing the independence between the pair, in curled brackets.

| **Independence Claim** | **Model^a^** | **p-value** |
| --- | --- | --- |
| (MFS, ForageGrass)\|{Crop} | MFS ~ Crop + ForageGrass | 0.30058 |
| (Forest, MFS)\|{Crop, ForageGrass, Shrub} | Forest ~ Crop + ForageGrass + Shrub + MFS | 0.0241 |
| (Edge, MFS)\|{Crop, Forest, ForageGrass, Shrub} | Edge ~ Crop + Forest + ForageGrass + Shrub + MFS | 0.20233 |
| (Shrub, MFS)\|{Crop, ForageGrass, Forest} | Shrub ~ Crop + ForageGrass + Forest + MFS | 0.24 |
| (RichShrub, MFS)\|{Crop, Edge, Shrub, HR} | RichShrub ~ Crop + Edge + Shrub + HR + MFS | 0.9774 |
| (HR, Forest)\|{Crop, ForageGrass, MFS, Shrub} | HR ~ Crop + ForageGrass + MFS + Shrub + Forest | 0.031856 |
| (RichShrub, Forest)\|{Crop, ForageGrass, Edge, Shrub, HR} | RichShrub ~ Crop + ForageGrass + Edge + Shrub + HR + Forest | 0.5673 |
| (Edge, HR)\|{Forest, Crop, ForageGrass, MFS, Shrub} | Edge ~ Forest + Crop + ForageGrass + MFS + Shrub + HR | 0.73213 |
| (Shrub, HR)\|{Crop, ForageGrass, MFS, Forest} | Shrub ~ Crop + ForageGrass + MFS + Forest + HR | 0.632 |

*Notes:* MFS = mean field size, ForageGrass = perennial forages and grassland, Crop = cropland, Shrub = shrubland, Edge = forest edge, RichShrub = shrub-edge bird richness, HR = Hedgerow. ^a^ Relationships with the RichShrub response variable were tested using generalized linear models with a Poisson distribution. Relationships between all other variables were tested using linear models.

**Table S12** Coefficient estimates, AICc, and ∆AICc values for perennial forages and grassland (Figure 2 – 4) modelled on its candidate set of standardized predictors measured in 1 km^2^ landscapes surrounding bird survey sites. The bolded model represents the top model.

| **Model** | **Crop Estimate** | **AICc** | **∆AIC** |
| --- | --- | --- | --- |
| **Crop** | **-0.22** | **359.95** | 0 |
| null |  | 364.41 | 4.46 |

**Table S13** Coefficient estimates, AICc, and ∆AICc values for mean field size (Figure 2 – 4) modelled on its candidate set of standardized predictors measured in 1 km^2^ landscapes surrounding bird survey sites. The bolded model represents the top model.

| **Model** | **Crop Estimate** | **AICc** | **∆AIC** |
| --- | --- | --- | --- |
| **Crop** | **0.60** | **309.57** | 0 |
| null |  | 364.41 | 54.84 |

**Table S14** Forest bird model coefficient estimates, AICc, and ∆AICc values for the amount of forest (Figure 2 - 3) modelled on its candidate set of standardized predictors measured in 1 km^2^ landscapes surrounding bird survey sites. The bolded model represents the top model.

| **Model** | **Crop Estimate** | **ForageGrass Estimate** | **AICc** | **∆AIC** |
| --- | --- | --- | --- | --- |
| **Crop + ForageGrass** | **-0.81** | **-0.63** | **146.87** | 0 |
| ForageGrass |  |  | 338.16 | 191.29 |
| Crop |  |  | 290.21 | 143.33 |
| null |  |  | 364.41 | 217.54 |

**Table S15** Coefficient estimates, AICc, and ∆AICc values for the proportion of hedgerows (Figure 2 – 4) modelled on its candidate set of standardized predictors measured in 1 km^2^ landscapes surrounding bird survey sites. The bolded model represents the top model.

| **Model** | **Crop Estimate** | **ForageGrass Estimate** | **MFS Estimate** | **AICc** | **∆AIC** |
| --- | --- | --- | --- | --- | --- |
| **GLOBAL** | **0.52** | **0.52** | **-0.40** | **315.14** | **0** |
| Crop + ForageGrass |  |  |  | 331.69 | 16.55 |
| ForageGrass |  |  |  | 341.36 | 26.22 |
| MFS + ForageGrass |  |  |  | 342.03 | 26.89 |
| MFS + Crop |  |  |  | 354.61 | 39.47 |
| Crop |  |  |  | 362.80 | 47.66 |
| MFS |  |  |  | 364.62 | 49.49 |
| null |  |  |  | 364.41 | 49.27 |

**Table S16** Forest bird model coefficient estimates, AICc, and ∆AICc values for forest bird richness (Figure 2) modelled on its candidate set of standardized predictors measured in 1 km^2^ landscapes surrounding bird survey sites. The bolded model represents the top model.

| **Model** | **Crop Estimate** | **Forest Estimate** | **HR Estimate** | **AICc** | **∆AIC** |
| --- | --- | --- | --- | --- | --- |
| **Forest** |  | **0.32** |  | **644.81** | 0 |
| Forest + HR |  |  |  | 645.44 | 0.63 |
| Forest + Crop |  |  |  | 646.16 | 1.36 |
| GLOBAL |  |  |  | 647.13 | 2.32 |
| HR + Crop |  |  |  | 686.93 | 42.12 |
| Crop |  |  |  | 694.89 | 50.08 |
| HR |  |  |  | 730.94 | 86.13 |
| null |  |  |  | 748.65 | 103.84 |

**Table S17** Coefficient estimates, AICc, and ∆AICc values for open country bird richness (Figure 4) modelled on its candidate set of standardized predictors measured in 1 km^2^ landscapes surround bird survey sites. The bolded model represents the top model.

| **Model** | **Crop Estimate** | **ForageGrass Estimate** | **HR Estimate** | **MFS Estimate** | **AICc** | **∆AIC** |
| --- | --- | --- | --- | --- | --- | --- |
| **proCrop + proForageGrass** | **0.34** | **0.45** |  |  | **370.56** | 0 |
| proForageGrass + proCrop + proHR |  |  |  |  | 372.52 | 1.96 |
| proCrop + proForageGrass + sdMPS |  |  |  |  | 372.55 | 1.99 |
| GLOBAL |  |  |  |  | 374.52 | 3.96 |
| ForageGrass + MFS |  |  |  |  | 381.63 | 11.07 |
| ForageGrass + MFS + HR |  |  |  |  | 381.88 | 11.32 |
| ForageGrass |  |  |  |  | 384.50 | 13.94 |
| ForageGrass + HR |  |  |  |  | 385.58 | 15.01 |
| MFS + HR |  |  |  |  | 397.52 | 26.96 |
| Crop + MFS + HR |  |  |  |  | 399.11 | 28.55 |
| Crop + HR |  |  |  |  | 399.20 | 28.64 |
| HR |  |  |  |  | 400.47 | 29.91 |
| Crop |  |  |  |  | 404.45 | 33.89 |
| Crop + MFS |  |  |  |  | 406.14 | 35.58 |
| MFS |  |  |  |  | 406.48 | 35.92 |
| null |  |  |  |  | 407.61 | 37.05 |

**Table S18** Shrub-edge bird model coefficient estimates, AICc, and ∆AICc values for proportion of forest (Figure 3) modelled on its candidate set of standardized predictors measured in 1 km^2^ landscapes surrounding bird survey sites. The bolded model represents the top model.

| **Model** | **Crop Estimate** | **ForageGrass Estimate** | **Shrub Estimate** | **AICc** | **∆AIC** |
| --- | --- | --- | --- | --- | --- |
| **GLOBAL** | **-0.99** | **-0.64** | **-0.33** | **79.18** | 0 |
| Crop + ForageGrass |  |  |  | 146.87 | 67.69 |
| Crop + Shrub |  |  |  | 277.49 | 198.31 |
| Crop |  |  |  | 290.21 | 211.02 |
| ForageGrass + Shrub |  |  |  | 333.13 | 253.95 |
| ForageGrass |  |  |  | 338.16 | 258.97 |
| Shrub |  |  |  | 362.80 | 283.62 |
| Null |  |  |  | 364.41 | 285.23 |

**Table S19** Shrub-edge bird model coefficient estimates, AICc, and ∆AICc values for the amount of shrubland (Figure 3) modelled on its candidate set of standardized predictors measured in 1 km^2^ landscapes surrounding bird survey sites. The bolded model represents the top model.

| **Model** | **Crop Estimate** | **ForageGrass Estimate** | **Forest Estimate** | **AICc** | **∆AIC** |
| --- | --- | --- | --- | --- | --- |
| **GLOBAL** | **-1.61** | **-0.85** | **-1.29** | **254.11** | **0** |
| Crop + Forest |  |  |  | 307.32 | 53.21 |
| Crop |  |  |  | 320.04 | 65.92 |
| Crop + ForageGrass |  |  |  | 321.80 | 67.69 |
| Forest + ForageGrass |  |  |  | 360.39 | 106.28 |
| Forest |  |  |  | 362.80 | 108.69 |
| null |  |  |  | 364.41 | 110.30 |
| ForageGrass |  |  |  | 365.42 | 111.31 |

**Table S20** Shrub-edge bird model coefficient estimates, AICc, and ∆AICc values for the amount of forest edge (Figure 3) modelled on its candidate set of standardized predictors measured in 1 km^2^ landscapes surrounding bird survey sites. The bolded model represents the top model.

| **Model** | **Crop Estimate** | **ForageGrass Estimate** | **Forest Estimate*** | **Shrub Estimate** | **AIC** | **∆AIC** |
| --- | --- | --- | --- | --- | --- | --- |
| **GLOBAL** | **-0.48** | **-0.30** | **0.39** | **0.19** | **142.84** | **0.00** |
| Crop + ForageGrass + Forest |  |  |  |  | 151.31 | 8.47 |
| Crop + Forest + Shrub |  |  |  |  | 153.89 | 11.05 |
| Forest + Shrub |  |  |  |  | 154.31 | 11.48 |
| ForageGrass + Forest + Shrub |  |  |  |  | 156.13 | 13.30 |
| Crop + Forest |  |  |  |  | 186.14 | 43.30 |
| Crop + ForageGrass |  |  |  |  | 204.36 | 61.53 |
| Forest |  |  |  |  | 208.40 | 65.56 |
| Forest + ForageGrass |  |  |  |  | 210.03 | 67.19 |
| Crop + Shrub |  |  |  |  | 250.55 | 107.71 |
| Crop |  |  |  |  | 264.25 | 121.41 |
| ForageGrass + Shrub |  |  |  |  | 293.05 | 150.21 |
| Shrub |  |  |  |  | 309.45 | 166.61 |
| ForageGrass |  |  |  |  | 358.92 | 216.08 |
| null |  |  |  |  | 364.41 | 221.57 |

*Forest was modelled as a quadratic, the estimate is calculated by added the linear and quadratic estimates.

**Table S21** Shrub-edge model coefficient estimates, AICc, and ∆AICc values for shrub-edge bird richness (Figure 3) modelled on its candidate set of standardized predictors measured in 1 km^2^ landscapes surrounding bird survey sites. The bolded model represents the top model.

| **Model** | **Crop Estimate** | **ForageGrass Estimate** | **Edge Estimate** | **Shrub Estimate** | **HR Estimate** | **AIC** | **∆AIC** |
| --- | --- | --- | --- | --- | --- | --- | --- |
| **Crop + ForageGrass + Edge** | **0.21** | **0.13** | **0.20** |  |  | **781.69** | **0.00** |
| ForageGrass + Crop + Edge + Shrub |  |  |  |  |  | 782.25 | 0.56 |
| ForageGrass + Crop + Edge + HR |  |  |  |  |  | 782.36 | 0.67 |
| GLOBAL | 0.20 | 0.10 | 0.17 | 0.05 | 0.04 | 783.19 | 1.50 |
| Crop + Edge + HR |  |  |  |  |  | 785.28 | 3.59 |
| Crop + Edge + Shrub +HR |  |  |  |  |  | 785.45 | 3.76 |
| Crop + Shrub + HR |  |  |  |  |  | 785.82 | 4.13 |
| Crop + ForageGrass + Shrub |  |  |  |  |  | 786.27 | 4.58 |
| Crop + Shrub |  |  |  |  |  | 786.32 | 4.63 |
| HR |  |  |  |  |  | 786.53 | 4.84 |
| Shrub + HR |  |  |  |  |  | 786.72 | 5.03 |
| Crop + ForageGrass + Shrub + HR |  |  |  |  |  | 787.22 | 5.53 |
| Crop + Edge + Shrub |  |  |  |  |  | 787.34 | 5.65 |
| Edge + HR |  |  |  |  |  | 787.50 | 5.81 |
| null |  |  |  |  |  | 787.99 | 6.30 |
| Crop + HR |  |  |  |  |  | 788.10 | 6.41 |
| Crop + Edge |  |  |  |  |  | 788.28 | 6.59 |
| Shrub |  |  |  |  |  | 788.34 | 6.65 |
| ForageGrass + HR |  |  |  |  |  | 788.47 | 6.78 |
| Edge + Shrub + HR |  |  |  |  |  | 788.67 | 6.98 |
| ForageGrass + Shrub + HR |  |  |  |  |  | 788.71 | 7.02 |
| ForageGrass |  |  |  |  |  | 788.97 | 7.28 |
| Crop |  |  |  |  |  | 789.07 | 7.38 |
| ForageGrass + Edge + HR |  |  |  |  |  | 789.34 | 7.65 |
| Crop + ForageGrass |  |  |  |  |  | 789.45 | 7.76 |
| ForageGrass + Shrub |  |  |  |  |  | 789.50 | 7.81 |
| Crop + ForageGrass + HR |  |  |  |  |  | 789.82 | 8.13 |
| Edge |  |  |  |  |  | 789.84 | 8.15 |
| Edge + Shrub |  |  |  |  |  | 790.12 | 8.43 |
| ForageGrass + Edge |  |  |  |  |  | 790.53 | 8.84 |
| ForageGrass + Edge + Shrub + HR |  |  |  |  |  | 790.64 | 8.95 |
| ForageGrass + Edge + Shrub |  |  |  |  |  | 791.48 | 9.79 |
